# Aging and Environmental Exposures Alter Tissue-Specific DNA Methylation Dependent upon CpG Island Context

**DOI:** 10.1371/journal.pgen.1000602

**Published:** 2009-08-14

**Authors:** Brock C. Christensen, E. Andres Houseman, Carmen J. Marsit, Shichun Zheng, Margaret R. Wrensch, Joseph L. Wiemels, Heather H. Nelson, Margaret R. Karagas, James F. Padbury, Raphael Bueno, David J. Sugarbaker, Ru-Fang Yeh, John K. Wiencke, Karl T. Kelsey

**Affiliations:** 1 Department of Pathology and Laboratory Medicine, Brown University, Providence, Rhode Island, United States of America; 2 Department of Community Health, Center for Environmental Health and Technology, Brown University, Providence, Rhode Island, United States of America; 3 Department of Biostatistics, Harvard School of Public Health, Boston, Massachusetts, United States of America; 4 Department of Neurological Surgery, University of California San Francisco, San Francisco, California, United States of America; 5 Department of Epidemiology and Biostatistics, University of California San Francisco, San Francisco, California, United States of America; 6 Masonic Cancer Center, Division of Epidemiology and Community Health, University of Minnesota, Minneapolis, Minnesota, United States of America; 7 Section of Biostatistics and Epidemiology, Department of Community and Family Medicine, Dartmouth Medical School, Lebanon, New Hampshire, United States of America; 8 Department of Pediatrics, Women and Infants Hospital and Brown University, Providence, Rhode Island, United States of America; 9 Division of Thoracic Surgery, Brigham and Women's Hospital, Harvard Medical School, Boston, Massachusetts, United States of America; Friedrich Miescher Institute for Biomedical Research, Switzerland

## Abstract

Epigenetic control of gene transcription is critical for normal human development and cellular differentiation. While alterations of epigenetic marks such as DNA methylation have been linked to cancers and many other human diseases, interindividual epigenetic variations in normal tissues due to aging, environmental factors, or innate susceptibility are poorly characterized. The plasticity, tissue-specific nature, and variability of gene expression are related to epigenomic states that vary across individuals. Thus, population-based investigations are needed to further our understanding of the fundamental dynamics of normal individual epigenomes. We analyzed 217 non-pathologic human tissues from 10 anatomic sites at 1,413 autosomal CpG loci associated with 773 genes to investigate tissue-specific differences in DNA methylation and to discern how aging and exposures contribute to normal variation in methylation. Methylation profile classes derived from unsupervised modeling were significantly associated with age (*P*<0.0001) and were significant predictors of tissue origin (*P*<0.0001). In solid tissues (n = 119) we found striking, highly significant CpG island–dependent correlations between age and methylation; loci in CpG islands gained methylation with age, loci not in CpG islands lost methylation with age (*P*<0.001), and this pattern was consistent across tissues and in an analysis of blood-derived DNA. Our data clearly demonstrate age- and exposure-related differences in tissue-specific methylation and significant age-associated methylation patterns which are CpG island context-dependent. This work provides novel insight into the role of aging and the environment in susceptibility to diseases such as cancer and critically informs the field of epigenomics by providing evidence of epigenetic dysregulation by age-related methylation alterations. Collectively we reveal key issues to consider both in the construction of reference and disease-related epigenomes and in the interpretation of potentially pathologically important alterations.

## Author Summary

The causes and extent of tissue-specific interindividual variation in human epigenomes are underappreciated and, hence, poorly characterized. We surveyed over 200 carefully annotated human tissue samples from ten anatosites at 1,413 CpGs for methylation alterations to appraise the nature of phenotypically, and hence potentially clinically important epigenomic alterations. Within tissue types, across individuals, we found variation in methylation that was significantly related to aging and environmental exposures such as tobacco smoking. Individual variation in age- and exposure-related methylation may significantly contribute to increased susceptibility to several diseases. As the NIH–funded HapMap project is critically contributing to annotating the human reference genome defining normal genetic variability, our work raises key issues to consider in the construction of reference epigenomes. It is well recognized that understanding genetic variation is essential to understanding disease. Our work, and the known interplay of epigenetics and genetics, makes it equally clear that a more complete characterization of epigenetic variation and its sources must be accomplished to reach the goal of a complete understanding of disease. Additional research is absolutely necessary to define the mechanisms controlling epigenomic variation. We have begun to lay the foundations for essential normal tissue controls for comparison to diseased tissue, which will allow the identification of the most crucial disease-related alterations and provide more robust targets for novel treatments.

## Introduction

While all somatic cells in a given individual are genetically identical (excepting T and B cells), different cell types form highly distinct anatomic structures and carry out a wide range of disparate physiologic functions. The vast repertoire of cellular phenotypes is made possible largely via epigenetic control of gene expression, which is known to play a critical role in cellular differentiation. Epigenetics is the study of mitotically and/or meiotically heritable changes in gene function that cannot be explained by changes in DNA sequence [1], and includes critical normal processes such as X-chromosome inactivation and genomic imprinting. Alterations in epigenetic control have been linked to several human pathologic conditions including cancers, and Rett, ICF, and Beckwith-Wiedemann syndromes [2]–[5]. The most widely studied epigenetic mark is DNA cytosine methylation, most often investigated in the context of CpG dinculeotides in promoter regions which often have concentrations of CpGs known as CpG islands. Normal cells are thought to generally maintain CpG islands in an unmethylated state permissive to transcription [6]. However, emerging work has established the presence of tissue specific methylation patterns in normal tissue at these islands [7]–[10]. Further, just as normal genetic variation is now understood to be associated with a predisposition to a vast array of human diseases [11], it is important that we begin the research needed to define the underlying interindividual differences in tissue specific methylation that lead us to an understanding of the nature of the relationships that govern these crucial tissue specific differences.

We have previously distinguished normal and tumor tissues using methylation profiling [12],[13]. These studies demonstrated variability in the methylation profiles of pleural mesotheliomas and tumors of the head and neck that was, in part, attributable to etiologically important exposures. In a similar manner, there is a basic need for epigenetic profiling of normal tissues to more completely characterize the normal pattern of promoter methylation variation in development, aging, and in response to common environmental exposures such as alcohol and tobacco smoke.

Efforts to describe the methylation profiles of normal tissues are now underway. Recent genome-wide studies of methylation in normal human tissues have shown that DNA methylation profiles are tissue-specific and correlated with sequence elements [8]–[10], [14]–[16]. However, while these studies are groundbreaking in showing that tissues have different patterns of methylation, the underlying causes and extent of tissue-specific and non-specific interindividual variation in DNA methylation patterns remain largely unknown. In fact, in a follow up experiment from a larger effort, Illingworth *et al.* observed significant variation among individuals when bisulfite sequencing a particular CpG island, and suggested that larger-scale studies are required to determine the extent of interindividual variability in methylation patterns [9]. Epigenetic variation has been hypothesized to cause underlying differences in disease susceptibility among monozygotic twins, and young twin-pairs have been shown to be more epigenetically similar than older monozygotic twins [17]. Therefore, the aging process and differences in environment have been hypothesized to influence clinically significant changes in methylation profiles as individuals accumulate varying exposures with age. In fact, recent work has shown an overall trend of increased methylation associated with older age in normal human prostate and colon tissues in several genes [18],[19]. Although an increase in promoter methylation with aging is generally accepted, recent evidence from Bjornsson *et al.* suggests a more complex picture. These authors found both increased and decreased intra-individual global methylation levels (enriched for promoter regions) in peripheral blood cell DNA over time [20]. In this background, it is crucial to more extensively characterize the contribution of aging and the environment to tissue-specific interindividual epigenetic variation.

In this study we used Illumina's GoldenGate methylation platform to investigate cytosine methylation in 217 normal human tissue specimens from 10 different anatomic sites in order to begin to understand variation both between and within tissues across individuals. Profiling CpG methylation of normal human tissues allowed us to begin characterizing the role of aging and environmental exposures in interindividual methylation variation, as well as specific gene-loci determinant of normal tissue-specificity. This work highlights the dynamic nature of epigenomes, and begins to disentangle the roles of aging, environmental factors, and innate variability among individual epigenomic profiles, both within, and across tissues.

## Results

### Unsupervised clustering

**Figure 1:**
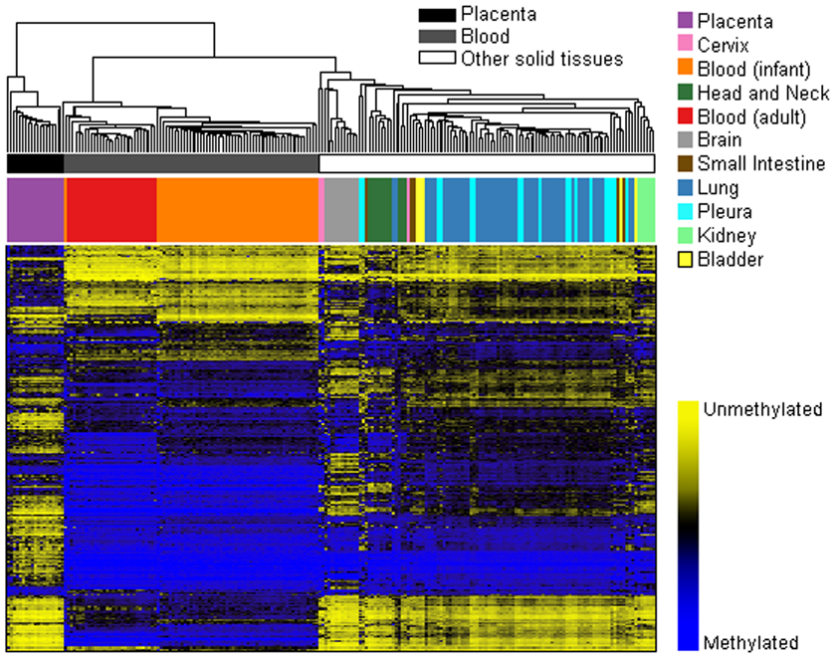
Unsupervised clustering of average beta values in normal human tissues. Normal tissue sample average beta values were subjected to unsupervised hierarchical clustering based on Manhattan distance and average linkage. Each column represents a sample and each row represents a CpG locus (500 most variable autosomal loci). Above the heatmap, colors indicate tissue type as in key. In the heat map blue = average β of one, or methylated, and yellow = average β of zero, or unmethylated.

**Figure 2:**
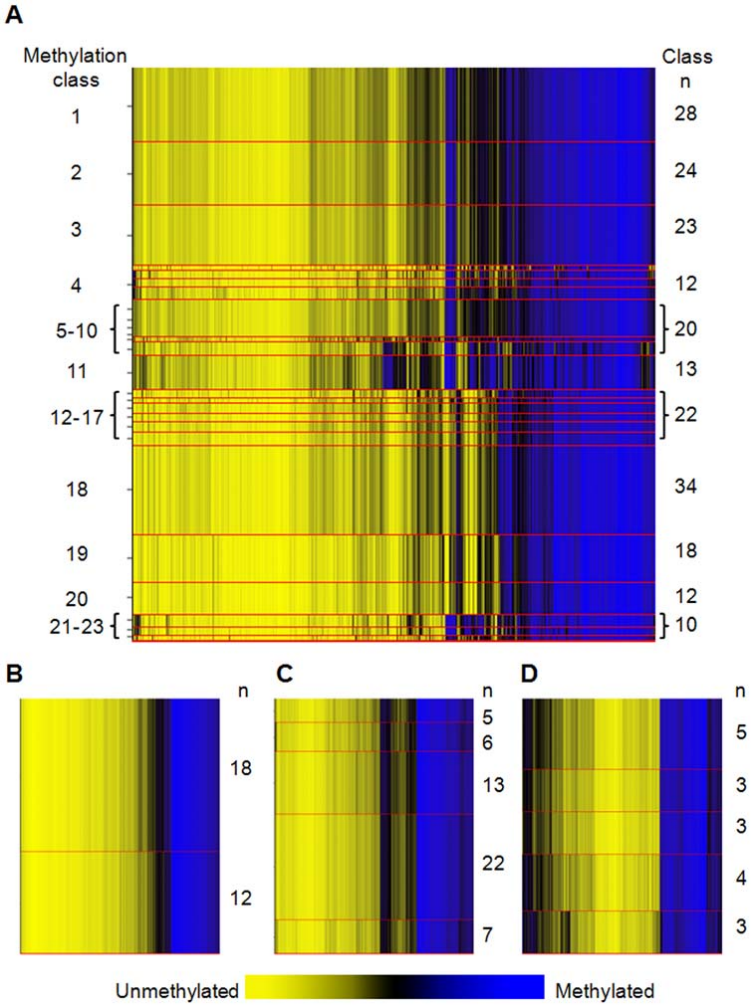
Recursive partitioning mixture models (RPMM) of methylation profiles in normal tissuses. Methylation average β is yellow for unmethylated and blue for methylated. Methylation profile classes are stacked in rows separated by red lines, and class height corresponds to the number of samples in each class. Class methylation at each CpG locus is a mean of methylation for all samples within a class. (A) Methylation profile classes significantly differentiate all normal tissue types (n = 217, *P*<0.0001). (B) Methylation profile classes from RPMM of adult bloods, class membership is significantly associated with age (*P*<0.005, n = 30). (C) Methylation profile classes from RPMM of lung tissue samples (n = 53). (D) Methylation profile classes from RPMM of pleural samples (n = 18).

Array methylation data were first assembled for exploration and visualization with unsupervised hierarchical clustering using Manhattan distance and average linkage for the 500 most variable autosomal CpG loci (Figure 1). Epigenetic profiles among these normal tissues are strikingly different. Applying recursively partitioned mixture modeling (RPMM) [21] to methylation data from all autosomal CpG loci across all 217 normal human tissue samples resulted in 23 methylation classes and their average methylation profiles (Figure 2A). Among the 23 classes in this model, 16 classes (70%) perfectly captured only a single tissue type (Table 1), and methylation profile classes were a highly significant predictor of sample tissue type (permutation *P*<0.0001). Further, age was significantly associated with methylation classes (*P*<0.0001). Separating samples into groups as placenta, blood, or other solid tissue, we found a significant association between group and methylation profile classes (*P*<0.0001).

**Table 1:** Recursively partitioned mixture model-derived CpG methylation profile class membership significantly predicts tissue type.

Methylation class #	Total n	n(%) Bladder	Adult Blood	Brain	Cervical	Infant blood	Head & Neck	Kidney	Lung	Placenta	Pleura	Small Intestine
1	28	3 (11)	-	-	-	-	-	2 (7)	12 (43)	-	8 (29)	3 (11)
2	24	-	-	-	-	-	-	-	19 (79)	-	5 (21)	-
3	23	-	-	-	-	-	-	-	20 (87)	-	2 (9)	1 (4)
4	2	-	-	-	**2 (100)**	-	-	-	-	-	-	-
5 - 6	6	-	-	-	-	-	**6 (100)**	-	-	-	-	-
7	5	-	-	1 (20)	-	-	4 (80)	-	-	-	-	-
4	12	2 (14)	-	2 (14)	1 (7)	-	-	2 (29)	2 (14)	-	2 (14)	1 (7)
9	2	-	-	-	-	-	-	-	-	1 (50)	1 (50)	-
10 - 11	18	-	-	-	-	-	-	-	-	**18 (100)**	-	-
12 - 18	56	-	-	-	-	**56 (100)**	-	-	-	-	-	-
19 - 20	30	-	**30 (100)**	-	-	-	-	-	-	-	-	-
21 -22	8	-	-	**8 (100)**	-	-	-	-	-	-	-	-
23	2	-	-	1 (50)	-	-	1 (50)	-	-	-	-	-

*: Permutation test for association between tissue type and methylation profile class ***P*** **<0.0001.**

### Supervised clustering

Random Forests (RF) classification of all samples based on methylation average beta values at all autosomal loci returned a confusion matrix showing: which samples are correctly classified, which are misclassified, and the misclassification error rate for each sample type (Table 2). Overall, 19 samples were confused with different tissue types, giving an overall misclassification error rate of 8.8%, significantly lower than expected under the null hypothesis (*P*<0.0001). Not unexpectedly, tissue types with larger sample sizes showed a significantly reduced misclassification error rates (*P*<0.05). The mean and standard deviation of average beta values for all autosomal CpG loci in each tissue type, and values for the decrease in random forest classification accuracy with locus removal are given in Table S1. In a RF analysis that examined whether samples could be correctly classified as placenta, blood, or other solid tissue, no samples were misclassified (misclassification error = 0%, *P*<0.0001), and the mean and standard deviation of average beta values for all autosomal CpG loci in each of placenta, blood, or solid tissue, and values for the decrease in random forest classification accuracy with locus removal are given in Table S2.

**Table 2:** Normal tissue CpG methylation-based random forests classification confusion matrix.

	Bladder	Adult blood	Brain	Cervix	Infant blood	Head & Neck	Kidney	Lung	Placenta	Pleura	Small intestine	Sample type n	Classification Error
Bladder	3	-	-	-	-	-	-	2	-	-	-	5	40%
Adult blood	-	30	-	-	-	-	-	-	-	-	-	30	0%
Brain	-	-	10	-	-	2	-	-	-	-	-	12	17%
Cervix	-	-	-	-	-	2	-	1	-	-	-	3	100%
Infant blood	-	-	-	-	55	-	-	-	-	-	-	55	0%
Head & Neck	-	-	-	-	-	10	-	1	-	-	-	11	9%
Kidney	-	-	-	-	-	-	5	1	-	-	-	6	17%
Lung	-	-	-	-	-	-	-	52	-	1	-	53	2%
Placenta	-	-	-	-	-	-	-	-	19	-	-	19	0%
Pleura	-	-	-	-	-	-	-	5	-	13	-	18	28%
Small intestine	-	-	-	-	-	-	-	4	-	-	1	5	80%

Overall misclassification error: 8.8% (*P*<0.0001)

### Tissue-specific RPMM

Variation in tissue-specific methylation relative to differences between tissue types was first explored visually. Scatter plots of methylation values for representative samples from two different tissues were less well correlated than similar plots of two representative samples from the same tissue type, though variation in tissue-specific methylation was also evident (Figure S1). Tissue-specific methylation patterns for adult blood, lung, and pleural tissue samples were then modeled with RPMM to investigate potential associations of age and exposures with methylation profiles. An RPMM of adult bloods (n = 30) resulted in two methylation classes (Figure 2B), and age differed significantly by methylation class (*P*<0.005), though we did not detect significant associations between methylation class and smoking status, packyears, or alcohol consumption. An RPMM of lung tissues (n = 53) resulted in five methylation classes (Figure 2C) where class membership was not associated with age or smoking status. An RPMM of pleural tissues (n = 18) resulted in five methylation classes (Figure 2D), and class membership was not associated with age; yet, an association between methylation class and asbestos exposure approached significance (*P*<0.07).

### Locus-by-locus analysis of exposure-related methylation

While exposures were not strongly associated with array-wide methylation profiles, locus-specific analysis revealed several exposure-related methylation alterations. Among pleural tissues 24 CpG loci had asbestos-related alterations in methylation, all of which were increases in methylation (*Q*<0.05, Table S3). In adult bloods, increasing packyears of smoking was significantly associated with *MLH1* (*Q*<0.0001), and *RIPK3* (*Q*<0.002) methylation; and over 30 CpG loci had significantly altered methylation in never versus ever drinkers (*Q*<0.05, Table S4). Among lung tissues, smoking status (never/ever) was associated with altered methylation at 138 CpG loci (*Q*<0.05, Table S5).

### CpG locus–specific, age-related methylation

Given our results from RPMM and previous reports of age-related increases in methylation in normal tissues [18],[19],[22] we next focused on age-related methylation at specific CpG loci. We began by examining gene-loci that other investigators have reported to be associated with age and found that *ESR1*, *GSTP1*, *IGF2*, *MGMT*, *MYOD1*, *RARB*, and *RASSF1* had significant age-associated methylation alterations, the majority of which were increases (*P*<0.05, age range >0, n = 139, Table 3). Hypothesizing that alterations in epigenetic regulatory genes or genes involved in aging processes could lead to the observed associations between age and methylation profiles from RPMM, we tested CpG loci in epigenetic regulatory genes, telomere maintenance genes, and a premature aging syndrome gene, again finding significant age related methylation alterations (Table 3). For example, *LAMB1* – involved in subchromosome domain positioning [23] – had increased methylation with age. Significant age-related methylation alterations in telomere maintenance gene-loci *TERT*, *ERCC1*, *RAD50*, and the Werner syndrome gene-locus (*WRN*) were also observed. Additionally, and in contrast to the predominantly increased age associated methylation at other gene-loci, there was a significant age-related decrease in CpG methylation of the *de novo* methyltransferase *DNMT3B*; and unlike the vast majority of other CpGs tested, *DNMT3B*_P352 was not located in a CpG island (Table 3).

**Table 3:** Significant age-related methylation alterations from selected CpG loci in normal tissues.

*GENE*_CpG	In CpG island	All Samples^a^	Solid Tissues	Lung	Blood	Pleura	Head & Neck	Brain
		n = 139	n = 110	n = 52^c^	n = 29^c^	n = 18	n = 11^c^	n = 10^d^
		*P*-value	*P*-value	*P*-value	*P*-value	*P*-value	*P*-value	*P*-value
*ESR1*_P151	yes	<0.01	<0.005	<0.001		<0.02		
*GSTP1*_E322	yes		<0.02					
*GSTP1*_P74	yes	<0.001						
*GSTP1*_seq	yes	<0.002						
*IGF2*_P36	yes	<0.05	<0.02					<0.04
*IGF2_E134*	yes					<0.02		<0.02^b^
*MGMT*_P281	yes	0.05^b^						
*MYOD1*_E156	yes					<0.02		
*MYOD1_P50*	yes							<0.01^b^
*RARB*_E114	yes	<0.05	<0.02					
*RARB*_P60	yes	0.05	<0.02					
*RASSF1*_E116	yes			<0.05^b^		<0.03		
*RASSF1*_P244	yes	<0.01^b^				<0.02		
*DNMT1*_P100	yes	<0.02^b^		<0.05^b^				
*DNMT3B*_P352	no			<0.0001^b^	<0.05^b^		<0.001^b^	
*HDAC1*_P414	yes	<0.01^b^	<0.05^b^					<0.01
*HDAC5*_E298	yes							<0.05^b^
*HDAC7*A_P344	no	<0.0001	<0.002					
*HDAC11*_P556	yes		<0.05					
*LAMB1*_E144	yes	<0.05	<0.01			<0.02		
*ERCC1*_P354	yes							<0.005^b^
*ERCC1*_P440	yes			<0.01^b^	<0.05^b^			<0.0001^b^
*RAD50*_P191	yes	<0.001	<0.05				<0.02^b^	
*TERT*_P360	yes					<0.03		
*WRN*_P969	yes	<0.0001	<0.0002					

a All samples with age range ≠ 0.

b Decreased methylation with increasing age.

c One sample.

d Two samples missing age data.

*: CpG loci tested, with no age association: *ESR1*_E298, *IGF2*_P1036, *MGMT*_P272, *SFRP1*_E398 & P157, *TERT*_E20, *WRN*_E57.

### Array-wide, locus-by-locus analysis of age-related methylation

To expand the examination of age-associated methylation alterations, we performed array-wide locus-by-locus analysis of CpGs. For all tissues (age range >0, n = 139), after correcting for multiple comparisons, over 300 CpG loci had age-related methylation alterations (*Q*<0.05, Table 4). Restricting analysis to solid tissues (n = 119) revealed over 250 CpG loci with age-related methylation alterations (*Q*<0.05, Table 4). Tissue-specific locus-by-locus analysis of age-related methylation was also performed (tissue types with n >10), detailed in Table S6.

**Table 4:** Summary statistics of global CpG locus-by-locus age vs. methylation association tests.

Sample type	n	Age range	Mean	SD	n loci *Q*<0.05*	Positive correlation n (%)	Negative correlation n (%)	n loci *P*<0.05
All samples^a^	139	14–89	60.1	16	295	120 (41)	175 (59)	424
Solid tissues	110	14–89	60	17.3	274	137 (50)	137 (50)	393
Brain^b^	10	14–52	34.5	11.1	38	6 (32)	13 (68)	269
Lung^c^	52	22–89	66.8	12.3	8	3 (38)	5 (62)	155
Head & Neck	11	49–80	66.2	7.9	1	1 (100)	-	93
Blood^c^	29	47–81	60	10.3	0	-	-	109
Pleura	18	38–77	58.3	11.3	0	-	-	146

*: False discovery rate corrected *P*-value.

a All samples with age range >0.

b Two samples.

c One sample missing age data.

There is now a considerable literature that suggests that genome structure affects both the initial placement of DNA methylation marks in development [24] as well as protecting silenced regions from being perturbed later in life [25]. To examine the possibility that genomic structure can affect the changes we observed in normal methylation, we assessed the potential effects of CpG island status on age-related methylation, with the hypothesis that there may be differential susceptibility to changes in DNA methylation in queried regions defined as canonical islands compared to those not in CpG islands. A CpG island is defined according to Takai and Jones [26], as a region of 200 bp with a GC content of >55% with an observed to expected ratio of CpG >0.65. This analysis used Generalized Estimator Equations (GEE), which are robust to within-person correlation and to the influence of aberrant observations [27], and estimated mean associations between age and methylation by CpG island status. Among all solid tissues (n = 119), the direction of correlation between age and methylation differed dependent upon whether the CpG was found in a CpG island. Loci in CpG islands had significantly positive correlations between methylation and aging, while loci not in CpG islands had significant losses of methylation with aging (*P* = 7.0E-04; Table 5). Similar trends were observed for other solid tissue types; age-related associations with methylation were significantly positive for loci in CpG islands for pleural tissues, and significantly negative for loci not in islands in brain tissues (Table 5). Interestingly, among adult blood samples, significantly negative correlations between age and methylation alterations were observed irrespective of CpG island status (*P* = 5.2E-05, Table 5).

**Table 5:** The direction of age-related methylation coefficients is dependent on CpG island status.

Tissue^a^	Age-related methylation				
CpG island status	estimate (CpGs)^b^		95% CI		*P*
Solid tissues (n = 119)					
Not in island	−0.021	(−2)	−0.040	−0.001	
In CpG island	0.024	(4)	0.009	0.039	7.0E-04
Adult blood (n = 29)					
Not in island	−0.026	(−3)	−0.040	−0.012	
In CpG island	−0.018	(−2)	−0.033	−0.003	5.2E-05
Lung (n = 52)					
Not in island	−0.020	(−2)	−0.042	0.001	
In CpG island	0.004	(1)	−0.015	0.024	0.16
Pleura (n = 18)					
Not in island	−0.002	(−0)	−0.036	0.032	
In CpG island	0.059	(11)	0.023	0.096	0.004
Head and Neck (n = 11)					
Not in island	−0.009	(−1)	−0.049	0.030	
In CpG island	0.025	(4)	−0.072	0.122	0.77
Brain (n = 10)					
Not in island	−0.068	(−6)	−0.117	−0.019	
In CpG island	0.051	(8)	−0.031	0.134	0.006

a Tissue n is for samples with available age data.

b Estimate is per decade (∼n CpGs altered/decade; positive indicates methylation with age, negative for loss of methylation with age).

**Figure 3:**
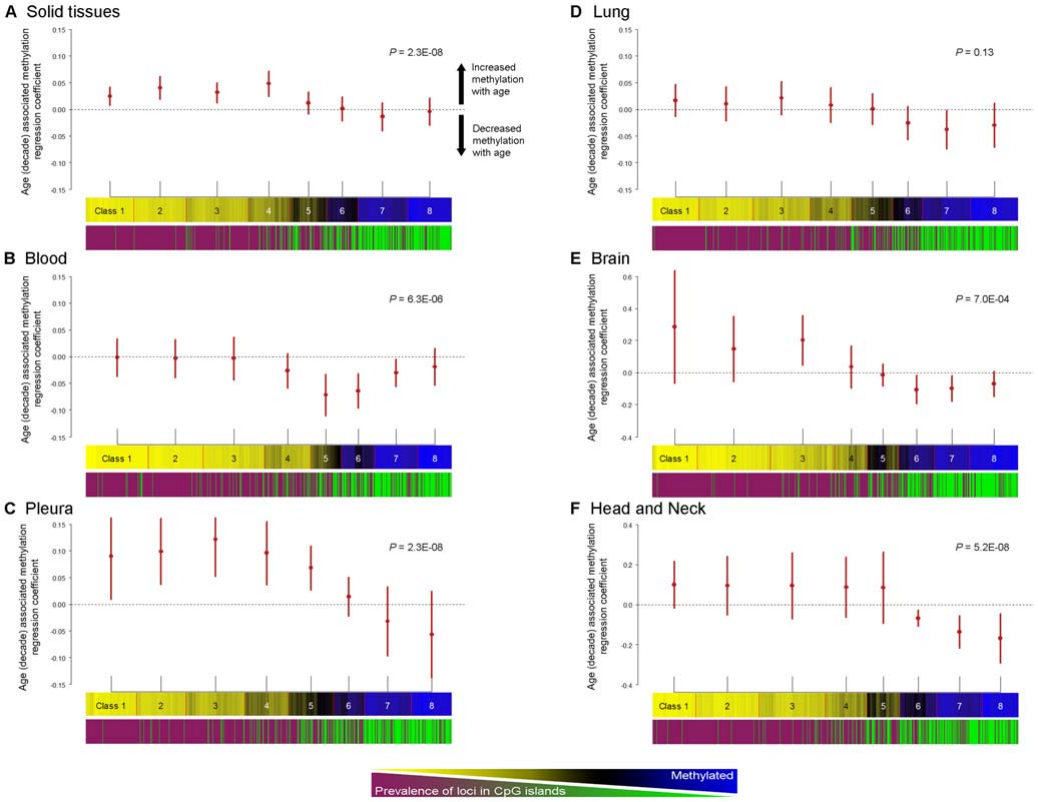
The direction of correlations for age associated methylation alterations differ dependent upon CpG island status. For (A–F), the top plot is the estimate of mean regression coefficients for age associated methylation (by decade), and its 95% confidence interval from GEE for each CpG RPMM class. The middle plot is of CpGs clustered with RPMM into eight classes for each group of samples. The bottom plot indicates the CpG island status for each locus (where magenta = CpG island locus, green = non-CpG island locus). (A) Estimates of class-specific age-associated methylation among all solid tissues (n = 119), RPMM clustering of CpGs, and CpG island status. Age-associated methylation is significantly increased among classes with a high prevalence of CpG-island loci (*P* = 2.3E-08). (B) Estimates of class-specific age-associated methylation among blood samples (n = 29), RPMM clustering of CpGs, and CpG island status. Age-associated methylation is significantly decreased among classes with a high prevalence of non-CpG-island loci (*P* = 6.3E-06). (C) Estimates of class-specific age associated methylation among pleural tissues (n = 18), RPMM clustering of CpGs, and CpG island status. Age-associated methylation is significantly increased among classes with a high prevalence of CpG-island loci (*P* = 2.3E-08). (D) Estimates of class-specific age-associated methylation among lung tissues (n = 52), RPMM clustering of CpGs, and CpG island status. (E) Estimates of class-specific age-associated methylation among brain tissues (n = 11), RPMM clustering of CpGs, and CpG island status. Age-associated methylation is significantly increased for the predominantly CpG island loci in class 3, and significantly decreased among classes with a high prevalence of non-CpG-island loci (*P* = 7.0E-04). (F) Estimates of class-specific age associated methylation among head and neck tissues (n = 10), RPMM clustering of CpGs, and CpG island status. Age-associated methylation is significantly decreased among classes with a high prevalence of non-CpG-island loci (*P* = 5.2E-08).

To investigate CpG-dependent correlations between aging and methylation in more detail we clustered CpGs (rather than samples) with RPMM (aiming to examine classes of CpGs with similar methylation profiles in more detail), grouping CpGs with similar methylation into eight separate classes. The CpG island status of all loci was plotted, and illustrates the well known tendency for CpGs located in islands to be unmethylated, while non-island CpGs tend to be methylated (Figure 3). We again used GEE, here estimating RPMM *class-specific* mean associations between age and methylation and plotted the estimates with their 95% confidence intervals. In a class-specific model for solid tissue samples, there was a positive correlation between age and methylation in classes whose loci were predominantly located in CpG islands (*P* = 1.9E-05, Figure 3A). The tissue specific analysis of pleura demonstrated that classes rich in CpG island loci had significant age-associated increases in methylation (*P* = 2.3E-08, Figure 3B). Interestingly, the pattern of class-specific correlations between age and methylation in adult bloods was similar to those for solid tissue types, though the correlation between age and methylation was shifted towards the negative such that there was a significant decrease in age-related methylation among loci not in CpG islands (*P* = 6.3E-06, Figure 3C). Lung tissues displayed a similar pattern of class-specific correlations between age and methylation, and the strength of these correlations approached statistical significance (*P* = 0.13, Figure 3D). Finally, both brain, and head and neck samples demonstrated increased age-associated methylation in classes rich in CpGs island loci, and decreases in age-associated methylation in classes rich in loci not in CpG islands (*P* = 7.0E-04, *P* = 5.2E-08, Figure 3E and 3F, respectively).

### Array validation and independent confirmation

**Figure 4:**
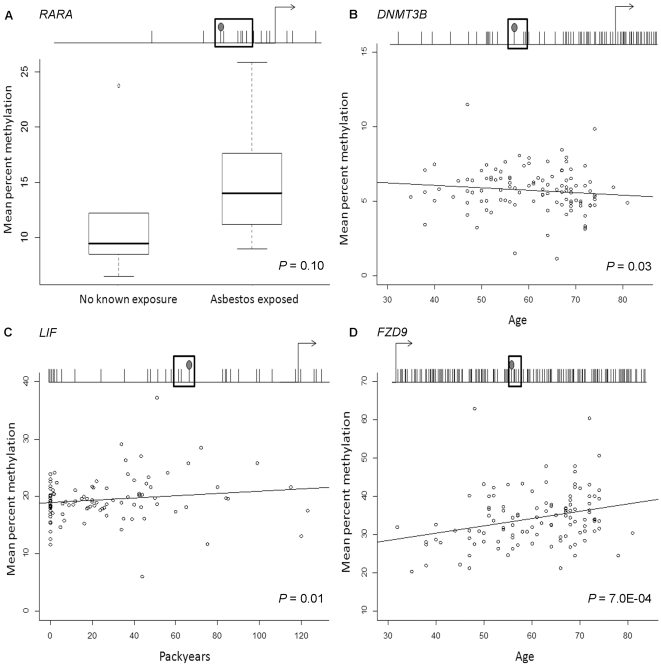
Bisulfite pyrosequencing confirmation of age and environmental exposure–related methylation alterations observed in array results. Each plot displays the gene transcription start site and surrounding CpGs are indicated by tick marks where the array CpG tick mark has a filled circle on top, and the sequenced CpGs are surrounded by a box. (A) Distribution of mean bisulfite pyrosequencing percent methylation for *RARA*_P176 and five downstream CpGs (79 bases total) stratified by known exposure to asbestos in DNA from pleural samples (n = 16) confirms the observation from array results of increased methylation at this locus among individuals with a known asbestos exposure (*P* = 0.10). (B) Mean bisulfite pyrosequencing percent methylation of *DNMT3B*_P352 and two downstream CpGs (30 bases total) in adult bloods run on the array and an independent set of controls plotted versus age (*P* = 0.03, rho = ––0.18, n = 112). (C) Mean bisulfite pyrosequencing percent methylation of *LIF*_P383 and two downstream CpGs (41 bases total) in adult bloods run on the array and an independent set of controls plotted versus smoking packyears (*P* = 0.01, rho = 0.24, n = 112). (D) Mean bisulfite pyrosequencing percent methylation of *FZD9*_E458 and five downstream CpGs (25 bases total) in adult bloods run on the array and an independent set of controls plotted versus age (*P* = 7.0E-04, rho = 0.29, n = 112).

Bisulfite modified DNA pyrosequencing was performed to validate array results. Array average beta values were significantly correlated with pyrosequencing percent methylation for sequenced array target CpGs; *RARA*_P176 (*P* = 0.003), *DNMT3B*_P352 (*P* = 0.008), and *LIF*_P383 (*P* = 3.0E-06, Figure S2). Consistent with array-based results, increased *RARA*_P176-local methylation was associated with reported asbestos exposure in pleural samples (n = 16, *P* = 0.10, Figure 4A). To confirm the observed associations between age or smoking packyears with methylation, specific loci were sequenced in array samples (n = 28) and an independent set of control blood DNAs (total n = 112). Sequencing *DNMT3B*_P352 both validated the association between decreased methylation and aging, and confirmed it in an independent population (*P* = 0.03, n = 112, Figure 4B). Similarly, the association between *LIF*_P383 methylation and packyears smoked from array results (*P*<0.02) was validated by pyrosequencing (*P*<0.02, n = 112, Figure 4C). In addition, pyrosequencing *FZD9*_E458-local CpGs confirmed the association between increased methylation and aging (*P*<0.001, n = 112, Figure 4D).

## Discussion

Epigenetic patterning and maintenance are of paramount importance for normal cellular functioning and identity. Hence, pursuing the annotation of normal human tissue-specific epigenomes is an important and necessary endeavor. However, such a project is considerably more challenging than sequencing the genome because of the tissue-specific and dynamic nature of epigenomes. Thus, a more complete understanding of what constitutes a normal epigenome, and the degree to which epigenomes vary (in a tissue dependent fashion) based on aging and the environment has the potential to dramatically improve the success of studies of epigenetic alterations in disease. Hence, our work characterized methylation of phenotypically important CpG loci across several human tissue types, elucidating interindividual tissue-specific variation in methylation profiles and the contribution of CpG island context to age associated methylation alterations. This work increases our appreciation for the dynamic nature of the epigenome, and begins to define basic tenets to follow in pursuit of both constructing reference epigenomes and elucidating epigenetic alterations truly indicative of disease states.

Using recursively-partitioned mixture modeling and random forests approaches, we differentiated tissues based on CpG methylation profile, consistent with other recent studies conducting genome-wide DNA methylation profiling [8]–[10],[15],[16]. These studies used high resolution methylation data and together have now shown that tissues have distinct methylation profiles. This novel and consistent body of work has, however, not addressed exposures in relation to interindividual variations in methylation. Not only do our findings confirm that tissue-specific epigenetic patterns can be readily defined with a targeted promoter-based CpG array, but they identify target sets of gene-loci most consistently capable of differentiating tissue types.

Factors known to contribute to methylation alterations include carcinogen exposures, inflammation, and diet. Several carcinogen exposures such as tobacco, alcohol, arsenic, and asbestos have been associated with methylation-induced gene-inactivation in various human cancers including bladder cancer, head and neck squamous cell carcinoma, and mesothelioma [28]–[33]. It is therefore reasonable to suggest that various and potentially accumulating exposures throughout life may directly or indirectly lead to methylation alterations and impact disease susceptibility. Carcinogens are well known to induce genetic abnormalities that can lead to clonal selection and expansion in normal appearing tissues (termed “field effect”). Hence, the association of carcinogen exposures with the occurrence of altered methylation at phenotypically important loci may arise as a consequence of altered (“initiated”) clones. Our data suggest that large epigenetic changes occur in normal appearing tissues, and the relationship of these changes to companion genetic changes is of interest to study in the future.

Cancer is a disease of aging, and initial studies of age-related methylation in normal tissues were motivated in large part by studies of methylation in cancer [34]. An early report from Issa *et al.* described an association between aging colonic mucosa and estrogen receptor methylation [35]. In general, trends of global (repeat element) hypomethylation and promoter hypermethylation found in cancer also have been observed in normal tissues with aging [36]. In recent reports of age-related methylation in normal human prostate and colon tissues, several CpG-island-containing genes were reported to have age-related increases in methylation [18],[19]. Our results confirm these findings and, in addition, document that age-related alterations in these CpG loci are tissue-dependent. More importantly, our examination of loci with previously reported age-associated methylation alterations, in conjunction with reports from others, suggested that the relationship between aging and promoter CpG methylation is complex. For example, using restriction-landmark genome scanning of over 2000 CpG loci in T lymphocytes comparing newborns, middle age, and elderly people, Tra *et al.* reported that 29 loci had age-related methylation alterations, with 23 loci displaying increased methylation with age and 6 decreasing with age [37]. In addition, measuring intra-individual global methylation changes over >10 years, Bjornsson *et al.* found both increased and decreased methylation levels dependent on the individual, with over 50% of participants exhibiting >5% change in methylation [20].

Stratifying our data on CpG-island status of loci, we showed that both the direction and strength of correlation between age and methylation were largely dependent upon CpG island status. More specifically, we found a propensity for CpG-island loci to gain methylation with age, and non-island CpGs to lose methylation with age. Our data are consistent with the literature that has demonstrated age-related increases in methylation at gene-loci found within CpG islands [18],[19],[22], as well as the findings of Tra *et al.* and Bjornsson *et al.* who showed bi-modal age-related methylation in normal tissues. A direct comparison, by examination of the data of Bjornsson *et al.*, indicated that a high percentage of their top 50 most age-altered loci (all decreases in methylation) are not located in CpG islands; among 24 of 30 autosomal CpGs in their Table 1 (where CpG island status can be identified by readers), only 5/24 (21%) are located in CpG islands, whereas 70% would be expected. Our results from blood samples corroborate their findings, and extend them to demonstrate similar trends in multiple other tissue types, where the strongest negative correlation between age and methylation occurs at CpGs which are not in CpG islands, and the strongest positive correlation between age and methylation occurs at loci in CpG islands.

The observed pattern of age associated methylation was irrespective of tissue-type, suggesting a common mechanism or dysregulation to explain these alterations. Reduced fidelity of maintenance methyltransferases with aging is one potential explanation for age related decreases in methylation; while age related increases in methylation could potentially reflect the accumulation of stochastic methylation events over time. As the examined tissues do not have a pathologic phenotype, methylated CpGs in these cells may not indicate dramatic functional consequences upon gene expression. However, the (in part selective) accumulation of alterations without readily detectable functional consequences should not be interpreted as biologically insignificant. Age-related drift of normal epigenomes without prominent changes in gene expression may nonetheless confer significantly increased risk of conversion to a pathologic phenotype by enhancing both the likelihood and frequency of methylation events that ultimately result in aberrant expression or altered genomic stability. For example, in the context of acquired “non-functional” CpG methylation in the promoter region of an aged individual, continued stochastic methylation events (e.g. “methylation spreading”) increase the chance of methylation induced silencing at that promoter (or silencing of another locus through action at a distance via silencing of other important regions such as enhancers), and hence, progression to a pathologic phenotype. Certainly, this hypothesis is especially plausible for the many diseases of aging. Alternatively, aberrant CpG methylation that silences a gene on a single allele may not appear to have a functional consequence if the complementary allele can provide compensatory expression. As a result, for example, clusters of cellular clones with mono-allelic gene expression could contribute to an increased risk of progression to a pathologic phenotype (e.g. loss of the 2^nd^ functional allele). Future population-based studies addressing the potential of quantifying age and/or exposure associated methylation alterations indicative of disease risk are necessary.

We have provided clear evidence of interindividual variation in tissue–specific methylation related to aging and environmental exposures at disease-relevant CpGs across 10 normal human tissue types. We have demonstrated both general and tissue specific alterations, uncovered a CpG island context-dependent directionality to age associated methylation alterations, and provided a novel path for examining the mechanistic basis of these alterations. By enumerating the methylation status of a panel of cancer-related genes known to stably control transcription in normal tissues, we have also afforded important controls for comparison to diseased tissues, potentially aiding in identification of the most critical alterations in specific diseases and providing more robust targets for novel treatments. Importantly, we have also begun to disentangle the contributions of aging and environmental factors to methylation alterations in normal tissues. Uncovering age and exposure-related methylation changes and their clear contextual dependence is an important contribution to our basic understanding of epigenetic maintenance as it relates to both aging and the pathologic process, provides a potential avenue to pursue clinically useful biomarkers, as well as to identify novel markers of disease susceptibility.

## Methods

### Study samples

Normal human tissues were assembled by a collaborative, multi-institutional network of investigators conducting molecular epidemiologic studies of human cancer. Tissues were obtained through Institutional Review Board approved studies already underway at these institutions, or were obtained from the National Disease Research Interchange (NDRI, Philadelphia, PA). Briefly, normal brain tissues (n = 12) were contributed by the Wiencke lab at UCSF through the San Francisco Adult Glioma Study [38]. Normal lung tissues (n = 49) were obtained from adjacent non-tumor portions of lung in patients treated for NSCLC [39] or from the NDRI from non-diseased individuals at autopsy (n = 4). Peripheral blood DNA was obtained from controls enrolled in a study of bladder cancer (n = 15) [40], controls enrolled in a study of head and neck cancer (n = 15) [41], and newborn infants (n = 55) [42]. Non-tumorigenic pleural samples (n = 18) were obtained from grossly disease-uninvolved regions of incident mesothelioma [28]. Head and neck anatomic sites (n = 11), bladder (n = 5), kidney (n = 6), and small intestine (n = 5) were obtained from the NDRI, all from individuals with no gross diseases or tumors of the obtained tissues. Non-pathologic placenta samples (n = 19) were obtained as residual tissues from control infant term births as part of an ongoing hospital-based case-control study of intrauterine growth restriction at Women and Infants Hospital in Providence RI. All tissues obtained from patients with disease (lung, pleura) were histologically confirmed as normal by independent study pathologist review of tissue samples prior to DNA extraction.

### Methylation analysis

Fresh frozen tissue and whole blood DNA was extracted using the QIAamp DNA mini kit according to the manufacturer's protocol (Qiagen, Valencia, CA). DNA was modified by sodium bisulfite to convert unmethylated cytosines to uracil using the EZ DNA Methylation Kit (Zymo Research, Orange, CA) according to the manufacturer's protocol. Illumina GoldenGate methylation bead arrays were used to simultaneously interrogate 1505 CpG loci associated with 803 cancer-related genes. Bead arrays have a similar sensitivity as quantitative methylation-specific PCR and were run at the UCSF Institute for Human Genetics, Genomics Core Facility according to the manufacturer's protocol and as described by Bibikova *et al* [43].

### Pyrosequencing

Quantification of cytosine percent methylation was performed by pyrosequencing bisulfite-converted DNA using the PyroMark MD pyrosequencing system (Biotage). Specific pyrosequencing primers were designed to amplify array CpG sites and as many downstream CpGs as conditions permitted (2 to 5 additional) using Biotage Assay Design Software v1.0.6.

All PCR reactions were carried out in 25 µl, utilized Qiagen Hot Star Taq polymerase, 5× Q solution (except *FZD9*), and 10× PCR buffer with 15 mM MgCl_2_ under the following conditions: 95°C 15′, (95°C 30″, 45°C (55°C for *RARA*) 30″, 72°C 1′) × 45 cycles, and 72°C 5′. Final reaction primer concentration for PCR and sequencing was 0.3 µM, primer details are in Table S7. All PCR reactions included a no template control, unmodified DNA control, and 7 standardized percent methylation controls (0%, 15%, 25%, 45%, 65%, 75%, and 100%) derived from Qiagen EpiTect PCR control DNA set samples. Sequencing reactions used 10 µl of PCR product and were run according to instrument/manufacturer protocols (Biotage).

### Statistical analysis

#### Data assembly

Data were assembled with BeadStudio methylation software from Illumina (SanDiego, CA). All array data points are represented by fluorescent signals from both methylated (Cy5) and unmethylated (Cy3) alleles, and methylation level is given by β = (max(*Cy5*, 0))/(|*Cy3*|+|*Cy5*|+100), the average methylation (β) value is derived from the ∼30 replicate methylation measurements. Raw average beta values were analyzed without normalization as recommended by Illumina. Each array CpG is annotated with the gene name followed by the CpG location (P for promoter, E for exonic, or (rarely) seq to reference a specific sequence) and its physical distance from the transcription start site. At each locus for each sample the detection *P*-value was used to determine sample performance; 5 samples (2%), had detection *P*-values >1.0E-05 at more than 25% of CpG loci and were removed from subsequent analysis. Similarly, CpG loci with a median detection *P*–value >0.05 (n = 8, 0.5%), were eliminated from analysis. Finally, all CpG loci on the X chromosome were excluded from analysis. The final dataset contained 217 samples and 1413 CpG loci associated with 773 genes. The manufacturer recommended CpG island designation of array CpGs was used and follows the definition of CpG island in [26].

#### Unsupervised clustering

Subsequent analyses were carried out using the R software [44]. For exploratory and visualization purposes, hierarchical clustering was performed using R function *hclust* with Manhattan metric and average linkage. To discern and describe the relationships between CpG methylation and tissue type (sample clustering), and the relationships between CpGs with coordinate methylation (CpG clustering), a modified model-based form of unsupervised clustering known as recursively partitioned mixture modeling (RPMM) was used as described in [21] and as used in [33]. This approach, a model-based version of HOPACH [45], and Dynamic Tree Cutting [46], built classes of samples based upon profiles of methylation with data using a mixture of beta distributions to recursively split samples into parsimoniously differentiated classes [47]–[50]. For sample clustering the number of classes was determined by recursively splitting the data via 2-class models with Bayesian information criterion (BIC) used at each potential split to decide whether the split was to be maintained or abandoned [21],[51],[52]. For CpG clustering the same approach was used and models were subsequently pruned to eight terminal classes. Permutation tests (running 10,000 permutations) were used to test for association with methylation class by generating a distribution of the test statistic for the null distribution for comparison to the observed distribution. For continuous variables, the permutation test was run with the Kruskal-Wallis test statistic. For categorical variables we used the standard chi-square statistic for testing association between two categorical variables.

#### Random forests

The R Package was also used to build classifiers with the Random Forest (RF) approach. RF is a tree-based classification algorithm similar to Classification and Regression Tree (CART) [53],[54] and was performed on CpG average beta values using RandomForest R package version 4.5–18 by Liaw and Wiener. RF builds each individual tree by taking a bootstrap sample (sampling with replacement) of the original data and on average about 1/3 of the original data are not sampled (out of bag or OOB). Those sampled are used as the training set to grow the trees (here 500,000), and the OOB data are used as the test set. At each node of the tree, a random sample of *m* out of the total *M* variables is chosen and the best split is found among the *m* variables. We utilized the default value for *m* in the Random Forest R package, √*M* (√1413 = 38). The misclassification error rate is the percentage of time the RF prediction is incorrect.

#### Locus-by-locus analysis

Associations between covariates and methylation at individual CpG loci were tested with a generalized linear model. The beta-distribution of average beta values was accounted for with a quasi-binomial logit link with an estimated scale parameter constraining the mean between 0 and 1, in a manner similar to that described by Hsuing *et al.* [55]. When testing for associations between age and CpG methylation, samples with an age range = 0 (placenta, infant bloods) were excluded. Tissue types with <10 samples (bladder, kidney, and small intestine) were not analyzed independently. CpG loci where an *a priori* hypothesis existed were tested separately, such as those that have been previously associated with aging in normal tissues [18],[19], as well as genes involved in epigenetic regulation, telomere maintenance (selected with GO term lists), and the premature aging syndrome (Werner) gene *WRN*. Array-wide scanning for CpG loci associations with sample type or covariate used false discovery rate estimation and *Q*–values computed by the *qvalue* package in R [56].

To test the hypothesis that there are associations of age and exposure with methylation, we constructed measures of partial methylation sets, in analogy with global DNA methylation, which is measured on repeats [57]. Specifically, we hypothesize that only CpGs that have limited functional significance (within a tissue) are allowed to acquire methylation alterations , and that these events are stochastic; consequently, only CpGs whose methylation varies across specimens of the same type will show evidence of association with age or exposure, but individual CpG methylation, as a binary event with low individual probability, may demonstrate correlation too weak to detect and validate in a subsequent sample. On the other hand, average methylation over a defined set of CpGs will have increased power to measure the totality of such events with the set. To define such sets, we conducted RPMM clustering on the CpGs (rather than specimens) over all specimens of a defined tissue type, pruning the resulting tree to 8 classes. Within each class we averaged methylation values to form a single, CpG-class-specific partial methylation statistic. For each set, we fit a quasi-binomial model [55] expressing the logit mean partial methylation for each class as a linear function of age or exposure; standard errors were computed using generalized estimating equations (GEE) [27], from which confidence intervals were constructed. Furthermore, to properly account for multiple comparisons in a statistically efficient manner, we tested the omnibus null hypothesis of no association in any of the 8 partial methylation classes using a Wald test statistic constructed from the GEE estimates and robust variance-covariance matrix. To test a similar methylation hypothesis while distinguishing between loci in CpG-Islands and those that are not in CpG islands, we followed a similar methodology, replacing the 8 CpG RPMM class designation with CpG island designation.

#### Pyrosequencing analysis

Sequencing data were processed using Pyro Q-CpG software v1.0.9 (Biotage) under default analysis parameters and exported for subsequent analysis in R software [44]. Associations between pyrosequencing percent methylation values and array average beta values or covariates such as age or environmental exposures were tested with a Spearman correlation test.

## Supporting Information

Figure S1 Pairwise plots comparing average beta values (A) between all blood and all head & neck samples, (B) individual blood sample versus an individual head & neck sample, comparisons within tissue type between individual samples for (C) blood and (D) head & neck. Average beta value scatterplots between tissue types indicate significant differences between tissues, and scatterplots within tissue type indicate relative similarity in the presence of interindividual variation. A) Mean of average betas for all blood samples (n = 30) versus mean of average betas for all head and neck samples (n = 11), indicates relatively high variability between tissue types, R2 = 0.84. B) Representative blood sample 1 average betas versus representative head and neck sample 1 average betas indicate similarly high variability between tissue types at the individual sample level, R2 = 0.87. C) Representative blood sample 2 versus representative blood sample 3 indicates relative similarity between individuals within a tissue type in the presence of interindividual variation, R2 = 0.97. D) Representative head and neck sample 1 versus representative head and neck sample 2 indicates relative similarity between individuals within a tissue type in the presence of interindividual variation, R2 = 0.96.
(0.07 MB TIF)

Figure S2 Bisulfite pyrosequencing mean percent methylation across all CpGs measured for RARA, DNMT3B, and LIF versus their respective CpG of interest on the array. Bisulfite pyrosequencing mean percent methylation across all CpGs measured for RARA, DNMT3B, and LIF versus their respective CpG of interest on the array. A) Mean bisulfite pyrosequencing percent methylation across array target CpG RARA_P176 and 5 downstream CpGs plotted versus Illumina GoldenGate methylation array average beta demonstrates a significant correlation between sequencing and array methylation (P = 0.03; n = 16). B) Mean bisulfite pyrosequencing percent methylation across array target CpG DNMT3B_P352 and 2 downstream CpGs plotted versus Illumina GoldenGate methylation array average beta demonstrates a significant correlation between sequencing and array methylation (P = 0.02; n = 28). Mean bisulfite pyrosequencing percent methylation across array target CpG LIF_P383 and 2 downstream CpGs plotted versus Illumina GoldenGate methylation array average beta demonstrates a significant correlation between sequencing and array methylation (P = 7.7E-08; n = 28).
(0.04 MB TIF)

Table S1 Autosomal CpG locus mean (sd) of average beta and values for the decrease in random forest classification accuracy with locus removal, all tissues.
(0.68 MB XLS)

Table S2 Autosomal CpG locus mean (sd) of average beta and values for the decrease in random forest classification accuracy with locus removal, for solid tissues, blood, and placenta.
(0.29 MB XLS)

Table S3 CpG loci with significantly altered methylation by reported asbestos exposure in pleural samples (n = 18).
(0.04 MB DOC)

Table S4 CpG loci with significantly altered methylation in never versus ever alcohol drinkers in blood (n = 29).
(0.05 MB DOC)

Table S5 CpG loci with significantly altered methylation by smoking in lung tissue (n = 53).
(0.13 MB DOC)

Table S6 Top 100 CpG loci associated with age by tissue type.
(0.84 MB DOC)

Table S7 Pyrosequencing assay primers.
(0.05 MB DOC)

## References

[1] Russo V, Martienssen RA, Riggs AD (1996). Epigenetic mechanisms of gene regulation..

[2] Feinberg AP, Tycko B (2004). The history of cancer epigenetics.. Nat Rev Cancer.

[3] Amir RE, Van den Veyver IB, Wan M, Tran CQ, Francke U (1999). Rett syndrome is caused by mutations in X-linked MECP2, encoding methyl-CpG-binding protein 2.. Nat Genet.

[4] Xu GL, Bestor TH, Bourc'his D, Hsieh CL, Tommerup N (1999). Chromosome instability and immunodeficiency syndrome caused by mutations in a DNA methyltransferase gene.. Nature.

[5] DeBaun MR, Niemitz EL, McNeil DE, Brandenburg SA, Lee MP (2002). Epigenetic alterations of H19 and LIT1 distinguish patients with Beckwith-Wiedemann syndrome with cancer and birth defects.. Am J Hum Genet.

[6] Jones PA, Baylin SB (2002). The fundamental role of epigenetic events in cancer.. Nat Rev Genet.

[7] Shiota K (2004). DNA methylation profiles of CpG islands for cellular differentiation and development in mammals.. Cytogenet Genome Res.

[8] Eckhardt F, Lewin J, Cortese R, Rakyan VK, Attwood J (2006). DNA methylation profiling of human chromosomes 6, 20 and 22.. Nat Genet.

[9] Illingworth R, Kerr A, Desousa D, Jorgensen H, Ellis P (2008). A novel CpG island set identifies tissue-specific methylation at developmental gene loci.. PLoS Biol.

[10] Rakyan VK, Down TA, Thorne NP, Flicek P, Kulesha E (2008). An integrated resource for genome-wide identification and analysis of human tissue-specific differentially methylated regions (tDMRs).. Genome Res.

[11] McCarthy MI, Hirschhorn JN (2008). Genome-wide association studies: potential next steps on a genetic journey.. Hum Mol Genet.

[12] Christensen BC, Houseman EA, Godleski JJ, Marsit CJ, Longacker JL (2009). Epigenetic profiles distinguish pleural mesothelioma from normal pleura and predict lung asbestos burden and clinical outcome.. Cancer Res.

[13] Marsit CJ, Christensen BC, Houseman EA, Karagas MR, Wrensch MR (2009). Epigenetic profiling reveals etiologically distinct patterns of DNA methylation in head and neck squamous cell carcinoma.. Carcinogenesis.

[14] Bock C, Paulsen M, Tierling S, Mikeska T, Lengauer T (2006). CpG island methylation in human lymphocytes is highly correlated with DNA sequence, repeats, and predicted DNA structure.. PLoS Genet.

[15] Rakyan VK, Hildmann T, Novik KL, Lewin J, Tost J (2004). DNA methylation profiling of the human major histocompatibility complex: a pilot study for the human epigenome project.. PLoS Biol.

[16] Schilling E, Rehli M (2007). Global, comparative analysis of tissue-specific promoter CpG methylation.. Genomics.

[17] Fraga MF, Ballestar E, Paz MF, Ropero S, Setien F (2005). Epigenetic differences arise during the lifetime of monozygotic twins.. Proc Natl Acad Sci U S A.

[18] Kwabi-Addo B, Chung W, Shen L, Ittmann M, Wheeler T (2007). Age-related DNA methylation changes in normal human prostate tissues.. Clin Cancer Res.

[19] Shen L, Kondo Y, Rosner GL, Xiao L, Hernandez NS (2005). MGMT promoter methylation and field defect in sporadic colorectal cancer.. J Natl Cancer Inst.

[20] Bjornsson HT, Sigurdsson MI, Fallin MD, Irizarry RA, Aspelund T (2008). Intra-individual change over time in DNA methylation with familial clustering.. JAMA.

[21] Houseman EA, Christensen BC, Marsit CJ, Karagas MR, Wrensch MR (2008). Model-based clustering of DNA methylation array data: a recursive-partitioning algorithm for high-dimensional data arising as a mixture of beta distributions.. BMC Bioinformatics.

[22] Issa JP (2003). Age-related epigenetic changes and the immune system.. Clin Immunol.

[23] Taddei A, Hediger F, Neumann FR, Gasser SM (2004). The function of nuclear architecture: a genetic approach.. Annu Rev Genet.

[24] Straussman R, Nejman D, Roberts D, Steinfeld I, Blum B (2009). Developmental programming of CpG island methylation profiles in the human genome.. Nat Struct Mol Biol.

[25] Esnault G, Majocchi S, Martinet D, Besuchet-Schmutz N, Beckmann JS (2009). Transcription factor CTF1 acts as a chromatin domain boundary that shields human telomeric genes from silencing.. Mol Cell Biol.

[26] Takai D, Jones PA (2002). Comprehensive analysis of CpG islands in human chromosomes 21 and 22.. Proc Natl Acad Sci U S A.

[27] Zeger SL, Liang KY (1986). Longitudinal data analysis for discrete and continuous outcomes.. Biometrics.

[28] Christensen BC, Godleski JJ, Marsit CJ, Houseman EA, Lopez-Fagundo CY (2008). Asbestos exposure predicts cell cycle control gene promoter methylation in pleural mesothelioma.. Carcinogenesis.

[29] Marsit CJ, Houseman EA, Schned AR, Karagas MR, Kelsey KT (2007). Promoter hypermethylation is associated with current smoking, age, gender and survival in bladder cancer.. Carcinogenesis.

[30] Marsit CJ, McClean MD, Furniss CS, Kelsey KT (2006). Epigenetic inactivation of the SFRP genes is associated with drinking, smoking and HPV in head and neck squamous cell carcinoma.. Int J Cancer.

[31] Toyooka S, Maruyama R, Toyooka KO, McLerran D, Feng Z (2003). Smoke exposure, histologic type and geography-related differences in the methylation profiles of non-small cell lung cancer.. Int J Cancer.

[32] Lyon CM, Klinge DM, Liechty KC, Gentry FD, March TH (2007). Radiation-induced lung adenocarcinoma is associated with increased frequency of genes inactivated by promoter hypermethylation.. Radiat Res.

[33] Christensen BC, Houseman EA, Godleski JJ, Marsit CJ, Longacker JL (2008). Epigenetic profiles distinguish pleural mesothelioma from normal pleura and predict lung asbestos burden and clinical outcome.. Cancer Res.

[34] Richardson B (2003). Impact of aging on DNA methylation.. Ageing Res Rev.

[35] Issa JP, Ottaviano YL, Celano P, Hamilton SR, Davidson NE (1994). Methylation of the oestrogen receptor CpG island links ageing and neoplasia in human colon.. Nat Genet.

[36] Fraga MF, Agrelo R, Esteller M (2007). Cross-talk between aging and cancer: the epigenetic language.. Ann N Y Acad Sci.

[37] Tra J, Kondo T, Lu Q, Kuick R, Hanash S (2002). Infrequent occurrence of age-dependent changes in CpG island methylation as detected by restriction landmark genome scanning.. Mech Ageing Dev.

[38] Wiemels JL, Wiencke JK, Sison JD, Miike R, McMillan A (2002). History of allergies among adults with glioma and controls.. Int J Cancer.

[39] Wiencke JK, Kelsey KT, Varkonyi A, Semey K, Wain JC (1995). Correlation of DNA adducts in blood mononuclear cells with tobacco carcinogen-induced damage in human lung.. Cancer Res.

[40] Karagas MR, Tosteson TD, Blum J, Morris JS, Baron JA (1998). Design of an epidemiologic study of drinking water arsenic exposure and skin and bladder cancer risk in a U.S. population.. Environ Health Perspect.

[41] Peters ES, McClean MD, Liu M, Eisen EA, Mueller N (2005). The ADH1C polymorphism modifies the risk of squamous cell carcinoma of the head and neck associated with alcohol and tobacco use.. Cancer Epidemiol Biomarkers Prev.

[42] Urayama KY, Wiencke JK, Buffler PA, Chokkalingam AP, Metayer C (2007). MDR1 gene variants, indoor insecticide exposure, and the risk of childhood acute lymphoblastic leukemia.. Cancer Epidemiol Biomarkers Prev.

[43] Bibikova M, Lin Z, Zhou L, Chudin E, Garcia EW (2006). High-throughput DNA methylation profiling using universal bead arrays.. Genome Res.

[44] R Development CT (2007). R: A Language and Environment for Statistical Computing..

[45] van der Laan M, Pollard K (2003). A new algorithm for hybrid hierarchical clustering with visualization and the bootstrap.. Journal of Statistical Planning and Inference.

[46] Langfelder P, Zhang B, Horvath S (2008). Defining clusters from a hierarchical cluster tree: the Dynamic Tree Cut package for R.. Bioinformatics.

[47] Shen L, Toyota M, Kondo Y, Lin E, Zhang L (2007). Integrated genetic and epigenetic analysis identifies three different subclasses of colon cancer.. Proc Natl Acad Sci U S A.

[48] Siegmund KD, Connor CM, Campan M, Long TI, Weisenberger DJ (2007). DNA methylation in the human cerebral cortex is dynamically regulated throughout the life span and involves differentiated neurons.. PLoS ONE.

[49] Siegmund KD, Laird PW, Laird-Offringa IA (2004). A comparison of cluster analysis methods using DNA methylation data.. Bioinformatics.

[50] Ji Y, Wu C, Liu P, Wang J, Coombes KR (2005). Applications of beta-mixture models in bioinformatics.. Bioinformatics.

[51] Fraley F, Raftery A (2002). Model-based clustering, discriminant analysis, and density estimation.. J Am Stat Assoc.

[52] Houseman EA, Coull BA, Betensky RA (2006). Feature-specific penalized latent class analysis for genomic data.. Biometrics.

[53] Breiman L (2001). Random Forests.. Machine Learning.

[54] Pico ASI, Chang JS, Yeh RF, Williamson DW, Wiemels JL, Wiencke JL, Tihan T, Conklin BR, Wrensch M.

[55] Hsiung DT, Marsit CJ, Houseman EA, Eddy K, Furniss CS (2007). Global DNA methylation level in whole blood as a biomarker in head and neck squamous cell carcinoma.. Cancer Epidemiol Biomarkers Prev.

[56] Storey J, Taylor J, Siegmund D (2004). Strong control, conservative point estimation, and simultaneous conservative consistency of false discovery rates: A unified approach.. J Royal Stat Soc Series B.

[57] Yang AS, Estecio MR, Doshi K, Kondo Y, Tajara EH (2004). A simple method for estimating global DNA methylation using bisulfite PCR of repetitive DNA elements.. Nucleic Acids Res.

