# Supplementary figures and images for: Aging and Environmental Exposures Alter Tissue-Specific DNA Methylation Dependent upon CpG Island Context
Source: PLoS Genet. 2009 Aug 14;5(8):e1000602. doi: 10.1371/journal.pgen.1000602 (PMC2718614; doi:10.1371/journal.pgen.1000602)

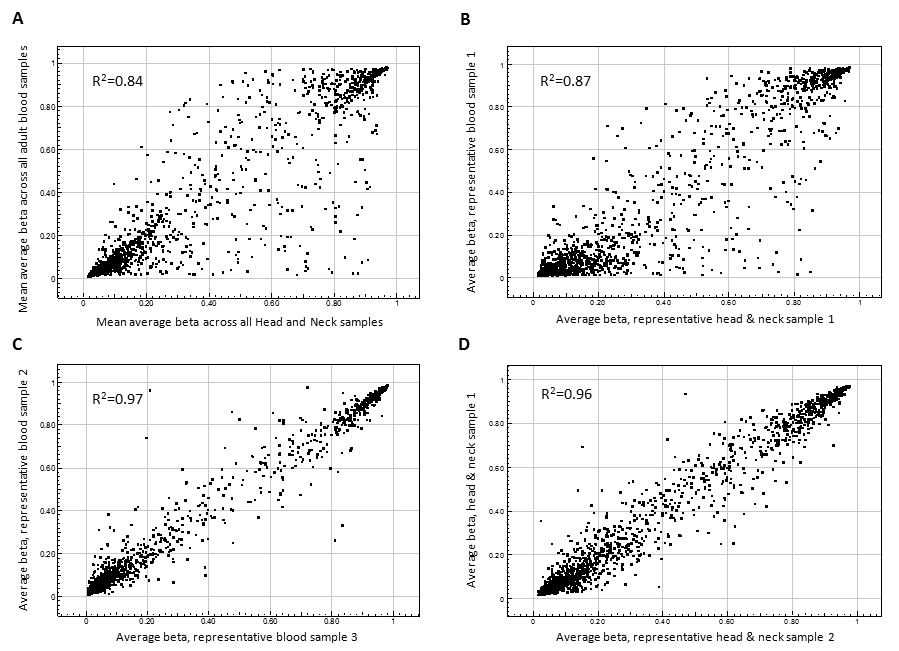

Supplement: Figure S1 — Pairwise plots comparing average beta values (A) between all blood and all head & neck samples, (B) individual blood sample versus an individual head & neck sample, comparisons within tissue type between individual samples for (C) blood and (D) head & neck. Average beta value scatterplots between tissue types indicate significant differences between tissues, and scatterplots within tissue type indicate relative similarity in the presence of interindividual variation. A) Mean of average betas for all blood samples (n = 30) versus mean of average betas for all head and neck samples (n = 11), indicates relatively high variability between tissue types, R2 = 0.84. B) Representative blood sample 1 average betas versus representative head and neck sample 1 average betas indicate similarly high variability between tissue types at the individual sample level, R2 = 0.87. C) Representative blood sample 2 versus representative blood sample 3 indicates relative similarity between individuals within a tissue type in the presence of interindividual variation, R2 = 0.97. D) Representative head and neck sample 1 versus representative head and neck sample 2 indicates relative similarity between individuals within a tissue type in the presence of interindividual variation, R2 = 0.96. (0.07 MB TIF) [file pgen.1000602.s001.tif]

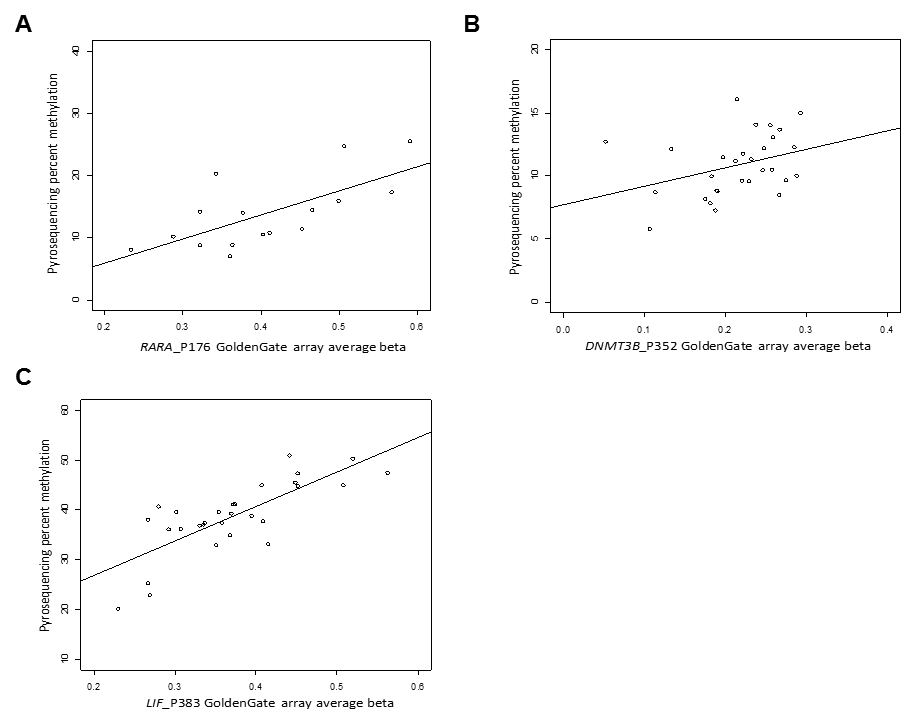

Supplement: Figure S2 — Bisulfite pyrosequencing mean percent methylation across all CpGs measured for RARA, DNMT3B, and LIF versus their respective CpG of interest on the array. Bisulfite pyrosequencing mean percent methylation across all CpGs measured for RARA, DNMT3B, and LIF versus their respective CpG of interest on the array. A) Mean bisulfite pyrosequencing percent methylation across array target CpG RARA_P176 and 5 downstream CpGs plotted versus Illumina GoldenGate methylation array average beta demonstrates a significant correlation between sequencing and array methylation (P = 0.03; n = 16). B) Mean bisulfite pyrosequencing percent methylation across array target CpG DNMT3B_P352 and 2 downstream CpGs plotted versus Illumina GoldenGate methylation array average beta demonstrates a significant correlation between sequencing and array methylation (P = 0.02; n = 28). Mean bisulfite pyrosequencing percent methylation across array target CpG LIF_P383 and 2 downstream CpGs plotted versus Illumina GoldenGate methylation array average beta demonstrates a significant correlation between sequencing and array methylation (P = 7.7E-08; n = 28). (0.04 MB TIF) [file pgen.1000602.s002.tif]
