# Supplementary material for: Aging and Environmental Exposures Alter Tissue-Specific DNA Methylation Dependent upon CpG Island Context
Source: PLoS Genet. 2009 Aug 14;5(8):e1000602. doi: 10.1371/journal.pgen.1000602 (PMC2718614; doi:10.1371/journal.pgen.1000602)
Supplement: Table S3 — CpG loci with significantly altered methylation by reported asbestos exposure in pleural samples (n = 18). (0.04 MB DOC) [file pgen.1000602.s005.doc]

Table S3. CpG loci with significantly altered methylation by reported asbestos exposure in pleural samples (n=18).

| *GENE* | CpG | Coefficient | *Q* - value |
| --- | --- | --- | --- |
| *PI3* | P274 | 0.67 | 0.014 |
| *PTCH2* | P568 | 0.53 | 0.014 |
| *CSF3R* | P472 | 0.48 | 0.017 |
| *MDS1* | E45 | 0.46 | 0.018 |
| *MKRN3* | P108 | 0.59 | 0.021 |
| *TRIP6* | P1090 | 0.79 | 0.023 |
| *INS* | P804 | 0.51 | 0.023 |
| *CASP10* | P334 | 0.71 | 0.025 |
| *PWCR1* | P811 | 0.50 | 0.030 |
| *SFTPA1* | E340 | 0.69 | 0.045 |
| *MKRN3* | E144 | 1.22 | 0.048 |
| *PWCR1* | E81 | 0.79 | 0.048 |
| *RARA* | P176 | 0.57 | 0.048 |
| *CEACAM1* | E57 | 0.69 | 0.048 |
| *SEPT9* | P374 | 0.70 | 0.048 |
| *ZIM3* | E203 | 0.49 | 0.048 |
| *NDN* | P1110 | 0.37 | 0.048 |
| *TRPM5* | P721 | 0.47 | 0.048 |
| *NID1* | P677 | 0.43 | 0.048 |
| *APOC1* | P406 | 0.30 | 0.048 |
| *ABL1* | P53 | 0.65 | 0.048 |
| *TMPRSS4* | P552 | 0.48 | 0.048 |
| *TIMP3* | P1114 | 0.42 | 0.048 |
| *MFAP4* | P197 | 0.41 | 0.049 |
