# Supplementary material for: Aging and Environmental Exposures Alter Tissue-Specific DNA Methylation Dependent upon CpG Island Context
Source: PLoS Genet. 2009 Aug 14;5(8):e1000602. doi: 10.1371/journal.pgen.1000602 (PMC2718614; doi:10.1371/journal.pgen.1000602)
Supplement: Table S4 — CpG loci with significantly altered methylation in never versus ever alcohol drinkers in blood (n = 29). (0.05 MB DOC) [file pgen.1000602.s006.doc]

| Table S4. CpG loci with significantly altered methylation in never versus ever alcohol drinkers in blood (n=29). | | | | | |
| --- | --- | --- | --- | --- | --- |
| *GENE* | CpG^a^ | *Q* - value | *GENE* | CpG^b^ | *Q* - value |
| *KRT5* | P308 | 0.000 | *ONECUT2* | P315 | 0.000 |
| *NDN* | P1110 | 0.000 | *MDR1* | S300 | 0.000 |
| *NBL1* | E205 | 0.000 | *HTR1B* | P222 | 0.000 |
| *TMPRSS4* | E83 | 0.001 | *HLA-F* | E402 | 0.001 |
| *PLG* | E406 | 0.005 | *DAB2* | P468 | 0.001 |
| *DDR1* | P332 | 0.005 | *MAF* | P826 | 0.001 |
| *SERPINA5* | E69 | 0.017 | *HS3ST2* | P171 | 0.003 |
| *AOC3* | P890 | 0.017 | *FRZB* | P406 | 0.005 |
| *GLI2* | P295 | 0.023 | *EFNA1* | P7 | 0.007 |
| *GLI3* | E148 | 0.026 | *OPCML* | E219 | 0.013 |
| *UGT1A1* | E11 | 0.028 | *LRP2* | E20 | 0.017 |
| *P2RX7* | P597 | 0.033 | *UNG* | P170 | 0.026 |
|  |  |  | *ABO* | E110 | 0.028 |
|  |  |  | *IGFBP3* | P423 | 0.028 |
|  |  |  | *NRG1* | E74 | 0.028 |
|  |  |  | *PROK2* | P390 | 0.028 |
|  |  |  | *EPO* | E244 | 0.038 |
|  |  |  | *IL1RN* | E42 | 0.044 |
|  |  |  | *ISL1* | E87 | 0.044 |
|  |  |  | *EXT1* | E197 | 0.048 |
| ^a^Increased methylation in ever, ^b^Increased methylation in never drinkers | | | | |  |
