# Supplementary material for: Aging and Environmental Exposures Alter Tissue-Specific DNA Methylation Dependent upon CpG Island Context
Source: PLoS Genet. 2009 Aug 14;5(8):e1000602. doi: 10.1371/journal.pgen.1000602 (PMC2718614; doi:10.1371/journal.pgen.1000602)
Supplement: Table S5 — CpG loci with significantly altered methylation by smoking in lung tissue (n = 53). (0.13 MB DOC) [file pgen.1000602.s007.doc]

Table S5. CpG loci with significantly altered methylation by smoking in lung tissue (n=53).

| *GENE* | CpG | Coefficient^a^ | *Q* - value |
| --- | --- | --- | --- |
| *ABO* | P312 | -0.52 | 0.000 |
| *CHGA* | E52 | -1.03 | 0.000 |
| *TNFRSF10A* | P91 | -0.81 | 0.000 |
| *MCAM* | P169 | -0.89 | 0.000 |
| *TBX1* | P520 | 0.64 | 0.001 |
| *INS* | P804 | 1.42 | 0.001 |
| *EPHA1* | E46 | -1.19 | 0.002 |
| *DHCR24* | P652 | 0.31 | 0.002 |
| *TNFRSF10A* | P171 | -0.80 | 0.004 |
| *CDKN1B* | P1161 | -0.53 | 0.004 |
| *NDN* | E131 | 0.37 | 0.005 |
| *GNAS* | P86 | 0.38 | 0.005 |
| *CTNNB1* | P757 | 0.40 | 0.005 |
| *VAV2* | E58 | -0.77 | 0.006 |
| *FAS* | P322 | -1.05 | 0.006 |
| *CTGF* | E156 | -0.67 | 0.006 |
| *TRPM5* | P721 | 0.61 | 0.006 |
| *PDE1B* | E141 | 0.37 | 0.006 |
| *HIC1* | P565 | -0.77 | 0.006 |
| *SNURF* | E256 | 0.40 | 0.006 |
| *TNFRSF1A* | P678 | 0.55 | 0.006 |
| *CCNC* | P132 | -0.82 | 0.006 |
| *SPI1* | P929 | 0.74 | 0.006 |
| *HLA-DOA* | P191 | 0.65 | 0.006 |
| *MALT1* | P406 | 0.49 | 0.006 |
| *LY6G6E* | P45 | 0.57 | 0.007 |
| *NFKB2* | P709 | -0.64 | 0.007 |
| *EPHA8* | P256 | 0.43 | 0.007 |
| *KRT13* | P676 | 0.81 | 0.008 |
| *JAG2* | P264 | -1.01 | 0.008 |
| *MLF1* | E243 | 0.41 | 0.009 |
| *TCF4* | P317 | 0.47 | 0.010 |
| *SNCG* | E119 | 0.72 | 0.012 |
| *XPC* | P226 | 0.28 | 0.013 |
| *FGFR4* | P610 | 0.44 | 0.013 |
| *RET* | S260 | -0.65 | 0.014 |
| *GLI3* | P453 | 0.42 | 0.015 |
| *ALPL* | P433 | 0.38 | 0.016 |
| *CDH17* | P376 | 0.38 | 0.016 |
| *PAX6* | P50 | -0.53 | 0.016 |
| *BMP3* | E147 | 0.35 | 0.016 |
| *IGF2AS* | E4 | 0.39 | 0.016 |
| *EPHA1* | P119 | -0.28 | 0.016 |
| *PROK2* | E0 | -0.40 | 0.016 |
| *MSH2* | P1008 | 0.39 | 0.016 |
| *STK11* | P295 | 0.52 | 0.016 |
| *CDKN1A* | P242 | -1.35 | 0.017 |
| *IPF1* | P750 | 0.69 | 0.017 |
| *SFTPC* | E13 | 0.43 | 0.017 |
| *GSTM2* | P453 | 0.67 | 0.017 |
| *INSR* | E97 | -0.57 | 0.017 |
| *SIN3B* | P514 | 0.38 | 0.017 |
| *KRT5* | P308 | 0.40 | 0.017 |
| *GML* | P281 | 0.50 | 0.017 |
| *ESR1* | S60 | 0.42 | 0.017 |
| *SLC5A8* | E60 | 0.51 | 0.017 |
| *NBL1* | E205 | 0.43 | 0.018 |
| *MBD2* | P233 | 0.59 | 0.020 |
| *MOS* | P746 | 0.47 | 0.020 |
| *TNC* | P57 | 0.50 | 0.020 |
| *SNURF* | P2 | 0.32 | 0.020 |
| *SYK* | P584 | 0.49 | 0.022 |
| *MEG3* | P235 | 0.30 | 0.022 |
| *TNC* | P198 | -0.43 | 0.022 |
| *NOTCH3* | P198 | 0.53 | 0.022 |
| *IRF7* | E236 | 0.43 | 0.022 |
| *FER* | E119 | 0.54 | 0.023 |
| *IL18BP* | E285 | 0.51 | 0.023 |
| *APBA2* | P227 | 0.30 | 0.023 |
| *ETS1* | E253 | -0.31 | 0.023 |
| *MMP19* | E274 | 0.38 | 0.023 |
| *AREG* | P217 | 0.67 | 0.024 |
| *MAP3K9* | E17 | -0.77 | 0.025 |
| *GAS7* | P622 | 0.45 | 0.025 |
| *COL18A1* | P494 | 0.50 | 0.025 |
| *LIG3* | P622 | 0.48 | 0.026 |
| *SKI* | E465 | -0.45 | 0.026 |
| *GABRB3* | P92 | -0.37 | 0.026 |
| *RAN* | P581 | 0.39 | 0.026 |
| *DDR1* | P332 | 0.71 | 0.026 |
| *SERPINA5* | E69 | 0.31 | 0.026 |
| *H19* | P1411 | 0.34 | 0.027 |
| *FGF9* | P862 | -0.27 | 0.027 |
| *P2RX7* | P597 | 0.44 | 0.028 |
| *MCAM* | P265 | 0.39 | 0.028 |
| *MXI1* | P75 | 0.52 | 0.028 |
| *DSC2* | E90 | 0.59 | 0.029 |
| *COL6A1* | P425 | 0.31 | 0.030 |
| *NOTCH3* | E403 | 0.52 | 0.030 |
| *NFKB1* | P496 | 0.41 | 0.030 |
| *TNFSF10* | E53 | -0.45 | 0.030 |
| *HRASLS* | P353 | 0.38 | 0.030 |
| *PTGS1* | P2 | -0.60 | 0.030 |
| *PDGFA* | P78 | 0.30 | 0.031 |
| *EPHA8* | P456 | 0.36 | 0.031 |
| *CALCA* | P171 | 0.43 | 0.031 |
| *IHH* | P529 | -0.51 | 0.031 |
| *NOS2A* | P288 | 0.43 | 0.032 |
| *BMPR1A* | P956 | -0.38 | 0.032 |
| *DDR2* | P743 | 0.47 | 0.032 |
| *DIRAS3* | E55 | 0.30 | 0.035 |
| *PKD2* | P336 | 0.31 | 0.035 |
| *PTGS2* | P524 | -0.65 | 0.036 |
| *HOXB2* | P99 | 0.41 | 0.036 |
| *FVT1* | P225 | -0.56 | 0.036 |
| *COL1A2* | P407 | 0.46 | 0.036 |
| *GSTM2* | P109 | 0.40 | 0.036 |
| *NGFR* | P355 | 0.37 | 0.036 |
| *GDF10* | E39 | -0.30 | 0.036 |
| *CPA4* | E20 | 0.53 | 0.036 |
| *CSPG2* | E38 | 0.44 | 0.036 |
| *NGFR* | E328 | 0.36 | 0.036 |
| *FGF5* | E16 | -0.40 | 0.038 |
| *RUNX1T1* | E145 | 0.57 | 0.038 |
| *ETV6* | E430 | -0.42 | 0.038 |
| *IL12B* | P392 | 0.93 | 0.038 |
| *MCC* | E23 | 0.32 | 0.038 |
| *TUSC3* | P85 | 0.39 | 0.038 |
| *ACVR1* | E328 | 0.46 | 0.038 |
| *MMP7* | P613 | 0.26 | 0.038 |
| *CTNNA1* | P185 | -0.40 | 0.038 |
| *BCR* | P346 | 0.38 | 0.039 |
| *CD1A* | P6 | 0.30 | 0.039 |
| *TDG* | E129 | 0.29 | 0.040 |
| *EPHA5* | P66 | 0.42 | 0.040 |
| *TRIP6* | P1274 | 0.45 | 0.041 |
| *FGF1* | P357 | 0.35 | 0.042 |
| *FGF8* | P473 | -0.61 | 0.042 |
| *IL3* | P556 | 0.32 | 0.046 |
| *PADI4* | P1158 | 0.46 | 0.046 |
| *SERPINE1* | E189 | 0.27 | 0.047 |
| *CYP2E1* | E53 | 0.34 | 0.047 |
| *P2RX7* | E323 | 0.44 | 0.047 |
| *SLC22A3* | P634 | 0.46 | 0.047 |
| *HBII-13* | E48 | 0.33 | 0.049 |
| *PXN* | P308 | 0.41 | 0.049 |
| *APBA1* | E99 | 0.37 | 0.049 |
| *CALCA* | E174 | 0.65 | 0.049 |
| ^a^Positive value for increased methylation in ever smokers versus never smokers | | | |
