# Supplementary material for: Aging and Environmental Exposures Alter Tissue-Specific DNA Methylation Dependent upon CpG Island Context
Source: PLoS Genet. 2009 Aug 14;5(8):e1000602. doi: 10.1371/journal.pgen.1000602 (PMC2718614; doi:10.1371/journal.pgen.1000602)
Supplement: Table S6 — Top 100 CpG loci associated with age by tissue type. (0.84 MB DOC) [file pgen.1000602.s008.doc]

| Table S6. Top 100 CpG loci associated with age by tissue type. | | | | | |
| --- | --- | --- | --- | --- | --- |
| All Samples | |  |  |  |  |
| Rank | *GENE* | CpG | Coefficient^a^ | *P -*value | *Q* - value |
| 1 | *FAS* | P65 | -0.21 | 1.00E-06 | 1.00E-06 |
| 2 | *CSF1* | P339 | -0.25 | 1.00E-06 | 1.00E-06 |
| 3 | *DNAJC15* | P65 | 0.21 | 1.00E-06 | 3.00E-06 |
| 4 | *CASP10* | P186 | -0.30 | 1.00E-06 | 6.00E-06 |
| 5 | *CDK2* | P330 | -0.21 | 1.00E-06 | 1.60E-05 |
| 6 | *IL8* | E118 | -0.25 | 1.00E-06 | 1.70E-05 |
| 7 | *CEACAM1* | E57 | -0.28 | 1.00E-06 | 2.00E-05 |
| 8 | *SHB* | P691 | -0.22 | 1.00E-06 | 2.60E-05 |
| 9 | *WRN* | P969 | 0.25 | 1.00E-06 | 2.60E-05 |
| 10 | *TNFRSF10D* | P70 | -0.25 | 1.00E-06 | 2.60E-05 |
| 11 | *EPHB6* | P827 | -0.08 | 1.00E-06 | 3.10E-05 |
| 12 | *DBC1* | E204 | 0.15 | 1.00E-06 | 6.40E-05 |
| 13 | *CD44* | P87 | -0.12 | 1.00E-06 | 6.40E-05 |
| 14 | *PYCARD* | E87 | -0.20 | 1.00E-06 | 6.60E-05 |
| 15 | *ACVR1C* | P363 | -0.16 | 1.00E-06 | 6.60E-05 |
| 16 | *SLC14A1* | E295 | -0.08 | 2.00E-06 | 8.30E-05 |
| 17 | *RARA* | P176 | 0.14 | 2.00E-06 | 8.80E-05 |
| 18 | *PLA2G2A* | E268 | -0.10 | 2.00E-06 | 8.80E-05 |
| 19 | *PRKCDBP* | E206 | -0.18 | 2.00E-06 | 8.80E-05 |
| 20 | *MST1R* | P392 | -0.26 | 2.00E-06 | 9.10E-05 |
| 21 | *PTHR1* | P258 | -0.12 | 3.00E-06 | 1.17E-04 |
| 22 | *CASP10* | E139 | -0.25 | 3.00E-06 | 1.20E-04 |
| 23 | *CD81* | P272 | 0.15 | 4.00E-06 | 1.32E-04 |
| 24 | *MKRN3* | P108 | 0.12 | 5.00E-06 | 1.63E-04 |
| 25 | *STAT5A* | E42 | -0.14 | 6.00E-06 | 1.97E-04 |
| 26 | *HDAC7A* | P344 | 0.19 | 8.00E-06 | 2.66E-04 |
| 27 | *HLA-DPA1* | P28 | -0.22 | 1.30E-05 | 3.91E-04 |
| 28 | *XPC* | P226 | 0.07 | 1.40E-05 | 4.14E-04 |
| 29 | *PSCA* | E359 | -0.20 | 1.60E-05 | 4.56E-04 |
| 30 | *ZAP70* | P220 | -0.12 | 1.70E-05 | 4.74E-04 |
| 31 | *TNFRSF1B* | P167 | -0.13 | 1.80E-05 | 4.74E-04 |
| 32 | *AIM2* | P624 | -0.18 | 2.10E-05 | 0.001 |
| 33 | *FZD9* | E458 | 0.13 | 2.10E-05 | 0.001 |
| 34 | *AIM2* | E208 | -0.10 | 2.90E-05 | 0.001 |
| 35 | *TNFSF10* | E53 | -0.20 | 4.00E-05 | 0.001 |
| 36 | *HLA-DPA1* | E35 | -0.18 | 4.30E-05 | 0.001 |
| 37 | *CDH11* | P354 | 0.11 | 4.50E-05 | 0.001 |
| 38 | *HGF* | P1293 | -0.05 | 5.10E-05 | 0.001 |
| 39 | *TSP50* | E21 | 0.10 | 5.40E-05 | 0.001 |
| 40 | *PLG* | P370 | -0.05 | 5.60E-05 | 0.001 |
| 41 | *SPP1* | P647 | -0.17 | 5.80E-05 | 0.001 |
| 42 | *VAV2* | P1182 | -0.10 | 7.00E-05 | 0.001 |
| 43 | *PTHLH* | P15 | -0.15 | 8.40E-05 | 0.002 |
| 44 | *VAMP8* | E7 | -0.13 | 8.90E-05 | 0.002 |
| 45 | *CASP10* | P334 | -0.14 | 1.09E-04 | 0.002 |
| 46 | *IL8* | P83 | -0.22 | 1.10E-04 | 0.002 |
| 47 | *IL10* | P348 | -0.14 | 1.15E-04 | 0.002 |
| 48 | *SOD3* | P225 | -0.10 | 1.20E-04 | 0.002 |
| 49 | *BCL2L2* | P280 | 0.11 | 1.26E-04 | 0.002 |
| 50 | *IRF7* | P277 | -0.14 | 1.26E-04 | 0.002 |
| 51 | *IL17RB* | E164 | 0.13 | 1.34E-04 | 0.002 |
| 52 | *ALPL* | P433 | -0.10 | 1.38E-04 | 0.002 |
| 53 | *SLC22A18* | P472 | -0.07 | 1.58E-04 | 0.002 |
| 54 | *MPO* | E302 | -0.11 | 1.61E-04 | 0.002 |
| 55 | *CCR5* | P630 | -0.06 | 1.66E-04 | 0.003 |
| 56 | *EMR3* | P39 | -0.20 | 1.78E-04 | 0.003 |
| 57 | *GSTM2* | P109 | -0.13 | 1.81E-04 | 0.003 |
| 58 | *BMPR1A* | P956 | 0.10 | 1.94E-04 | 0.003 |
| 59 | *DAPK1* | E46 | 0.07 | 1.94E-04 | 0.003 |
| 60 | *FOSL2* | E384 | 0.08 | 1.96E-04 | 0.003 |
| 61 | *APBA2* | P305 | -0.07 | 2.15E-04 | 0.003 |
| 62 | *TNFRSF1A* | P678 | -0.09 | 2.20E-04 | 0.003 |
| 63 | *IGFBP5* | P9 | 0.14 | 2.23E-04 | 0.003 |
| 64 | *CCKAR* | P270 | 0.09 | 2.50E-04 | 0.003 |
| 65 | *ZNF264* | P397 | 0.12 | 2.51E-04 | 0.003 |
| 66 | *WNT8B* | E487 | -0.06 | 2.75E-04 | 0.003 |
| 67 | *FAT* | P973 | 0.13 | 2.82E-04 | 0.004 |
| 68 | *MUC1* | P191 | -0.14 | 3.08E-04 | 0.004 |
| 69 | *AFF3* | P122 | -0.29 | 3.10E-04 | 0.004 |
| 70 | *HLA-DPA1* | P205 | -0.18 | 3.21E-04 | 0.004 |
| 71 | *IL6* | P213 | -0.13 | 3.22E-04 | 0.004 |
| 72 | *LTB4R* | P163 | -0.19 | 3.27E-04 | 0.004 |
| 73 | *RAD50* | P191 | 0.14 | 3.27E-04 | 0.004 |
| 74 | *TEK* | P479 | 0.09 | 3.32E-04 | 0.004 |
| 75 | *TJP2* | P518 | -0.16 | 3.35E-04 | 0.004 |
| 76 | *MPL* | P62 | -0.15 | 3.37E-04 | 0.004 |
| 77 | *IL10* | P85 | -0.17 | 3.47E-04 | 0.004 |
| 78 | *CASP8* | E474 | -0.16 | 3.50E-04 | 0.004 |
| 79 | *CCL3* | E53 | -0.10 | 3.58E-04 | 0.004 |
| 80 | *THPO* | E483 | -0.13 | 3.58E-04 | 0.004 |
| 81 | *MPO* | P883 | -0.16 | 3.67E-04 | 0.004 |
| 82 | *RAN* | P581 | 0.09 | 3.92E-04 | 0.004 |
| 83 | *PDGFB* | E25 | 0.08 | 4.06E-04 | 0.004 |
| 84 | *HOXB2* | P488 | 0.11 | 4.12E-04 | 0.004 |
| 85 | *CARD15* | P302 | -0.15 | 4.12E-04 | 0.004 |
| 86 | *APOA1* | P261 | 0.15 | 4.26E-04 | 0.004 |
| 87 | *LCK* | E28 | -0.07 | 4.37E-04 | 0.004 |
| 88 | *p16* | S188 | 0.09 | 4.43E-04 | 0.004 |
| 89 | *SOX17* | P287 | 0.13 | 4.44E-04 | 0.004 |
| 90 | *CD2* | P68 | -0.24 | 4.69E-04 | 0.004 |
| 91 | *DLC1* | E276 | -0.15 | 4.85E-04 | 0.004 |
| 92 | *PTHLH* | E251 | -0.12 | 5.06E-04 | 0.005 |
| 93 | *KLK11* | P1290 | -0.04 | 5.13E-04 | 0.005 |
| 94 | *SPDEF* | P6 | -0.10 | 5.22E-04 | 0.005 |
| 95 | *PLSCR3* | P751 | 0.07 | 5.24E-04 | 0.005 |
| 96 | *MAF* | P826 | 0.15 | 5.28E-04 | 0.005 |
| 97 | *PADI4* | E24 | -0.19 | 5.44E-04 | 0.005 |
| 98 | *DLL1* | P832 | 0.09 | 5.56E-04 | 0.005 |
| 99 | *SPI1* | P48 | -0.15 | 6.61E-04 | 0.006 |
| 100 | *SERPINE1* | E189 | -0.08 | 1.48E-04 | 0.001 |
| ^a^Change in average beta per decade | | |  |  |  |
|  |  |  |  |  |  |
| Solid tissues |  |  |  |  |  |
| Rank | *GENE* | CpG | Coefficient^a^ | *P -*value | *Q* - value |
| 1 | PLA2G2A | E268 | -0.12 | 1.00E-06 | 0.000 |
| 2 | FAS | P65 | -0.15 | 1.00E-06 | 0.000 |
| 3 | ONECUT2 | E96 | 0.12 | 1.00E-06 | 0.000 |
| 4 | CDKN2A | S188 | 0.11 | 1.00E-06 | 0.000 |
| 5 | AIM2 | E208 | -0.12 | 1.00E-06 | 0.000 |
| 6 | PTHR1 | P258 | -0.15 | 1.00E-06 | 0.000 |
| 7 | PYCARD | E87 | -0.14 | 2.00E-06 | 0.000 |
| 8 | HGF | P1293 | -0.06 | 6.00E-06 | 0.001 |
| 9 | WNT8B | E487 | -0.08 | 7.00E-06 | 0.001 |
| 10 | DNAJC15 | P65 | 0.15 | 9.00E-06 | 0.001 |
| 11 | CSF1 | P339 | -0.18 | 1.00E-05 | 0.001 |
| 12 | ACVR1C | P363 | -0.10 | 1.30E-05 | 0.001 |
| 13 | ACTG2 | P346 | -0.11 | 1.30E-05 | 0.001 |
| 14 | EPHB6 | P827 | -0.07 | 1.40E-05 | 0.001 |
| 15 | MAF | P826 | 0.18 | 1.40E-05 | 0.001 |
| 16 | DBC1 | E204 | 0.15 | 1.60E-05 | 0.001 |
| 17 | KLK11 | P1290 | -0.05 | 1.90E-05 | 0.001 |
| 18 | BMPR1A | P956 | 0.10 | 1.90E-05 | 0.001 |
| 19 | CASP10 | P186 | -0.17 | 2.00E-05 | 0.001 |
| 20 | PRKCDBP | E206 | -0.12 | 2.20E-05 | 0.001 |
| 21 | EGF | E339 | -0.17 | 2.30E-05 | 0.001 |
| 22 | CDK2 | P330 | -0.15 | 3.10E-05 | 0.001 |
| 23 | PALM2-AKAP2 | P420 | 0.12 | 3.60E-05 | 0.001 |
| 24 | CCR5 | P630 | -0.07 | 3.90E-05 | 0.001 |
| 25 | PLG | P370 | -0.06 | 4.50E-05 | 0.001 |
| 26 | EPHA7 | E6 | 0.12 | 4.60E-05 | 0.001 |
| 27 | CD44 | P87 | -0.11 | 5.00E-05 | 0.001 |
| 28 | RBP1 | P150 | 0.10 | 6.10E-05 | 0.002 |
| 29 | TNFRSF10D | P70 | -0.17 | 6.10E-05 | 0.002 |
| 30 | IL8 | E118 | -0.17 | 6.60E-05 | 0.002 |
| 31 | SHB | P691 | -0.13 | 7.00E-05 | 0.002 |
| 32 | SEMA3A | P343 | 0.13 | 7.50E-05 | 0.002 |
| 33 | CEACAM1 | E57 | -0.17 | 7.70E-05 | 0.002 |
| 34 | FZD9 | E458 | 0.13 | 8.00E-05 | 0.002 |
| 35 | COL4A3 | E205 | 0.07 | 9.10E-05 | 0.002 |
| 36 | RIPK1 | P868 | -0.14 | 1.30E-04 | 0.003 |
| 37 | IL17RB | E164 | 0.15 | 1.52E-04 | 0.003 |
| 38 | RARA | P176 | 0.12 | 1.93E-04 | 0.004 |
| 39 | WRN | P969 | 0.16 | 1.99E-04 | 0.004 |
| 40 | ZNFN1A1 | P179 | -0.06 | 2.12E-04 | 0.004 |
| 41 | CHD2 | P451 | -0.05 | 2.34E-04 | 0.004 |
| 42 | LOX | P313 | 0.10 | 2.57E-04 | 0.005 |
| 43 | ABCC5 | P444 | 0.07 | 2.64E-04 | 0.005 |
| 44 | PLSCR3 | P751 | 0.08 | 2.70E-04 | 0.005 |
| 45 | DCN | P1320 | -0.09 | 3.18E-04 | 0.005 |
| 46 | ABCB4 | E429 | -0.06 | 3.21E-04 | 0.005 |
| 47 | CDH11 | E102 | 0.08 | 3.38E-04 | 0.006 |
| 48 | MOS | P746 | -0.08 | 3.56E-04 | 0.006 |
| 49 | ZNF264 | P397 | 0.11 | 3.93E-04 | 0.006 |
| 50 | PDGFRA | E125 | 0.11 | 3.94E-04 | 0.006 |
| 51 | LEFTY2 | P719 | -0.09 | 3.96E-04 | 0.006 |
| 52 | PDGFB | E25 | 0.09 | 4.21E-04 | 0.006 |
| 53 | APBA2 | P305 | -0.08 | 4.38E-04 | 0.007 |
| 54 | AGTR1 | P154 | 0.16 | 4.55E-04 | 0.007 |
| 55 | SLC14A1 | E295 | -0.07 | 4.62E-04 | 0.007 |
| 56 | HOXB2 | P99 | 0.09 | 4.66E-04 | 0.007 |
| 57 | AGXT | E115 | -0.07 | 4.85E-04 | 0.007 |
| 58 | MMP3 | P55 | -0.08 | 4.89E-04 | 0.007 |
| 59 | TMEFF1 | P234 | 0.08 | 0.001 | 0.007 |
| 60 | SFTPC | E13 | -0.08 | 0.001 | 0.007 |
| 61 | NAT2 | P11 | -0.10 | 0.001 | 0.008 |
| 62 | FOSL2 | E384 | 0.08 | 0.001 | 0.008 |
| 63 | IGFBP3 | P1035 | 0.11 | 0.001 | 0.008 |
| 64 | IPF1 | P234 | 0.10 | 0.001 | 0.008 |
| 65 | TFF1 | P180 | -0.10 | 0.001 | 0.008 |
| 66 | SERPINE1 | E189 | -0.08 | 0.001 | 0.008 |
| 67 | ABCA1 | P45 | 0.10 | 0.001 | 0.008 |
| 68 | STAT5A | E42 | -0.12 | 0.001 | 0.008 |
| 69 | EDNRB | P709 | -0.06 | 0.001 | 0.008 |
| 70 | DAPK1 | E46 | 0.08 | 0.001 | 0.008 |
| 71 | IMPACT | P234 | 0.13 | 0.001 | 0.008 |
| 72 | MST1R | P392 | -0.15 | 0.001 | 0.008 |
| 73 | FLT3 | E326 | 0.16 | 0.001 | 0.009 |
| 74 | TMEFF2 | P152 | 0.06 | 0.001 | 0.010 |
| 75 | SOX17 | P287 | 0.13 | 0.001 | 0.010 |
| 76 | BDNF | P259 | 0.09 | 0.001 | 0.010 |
| 77 | RBP1 | E158 | 0.09 | 0.001 | 0.010 |
| 78 | PRDM2 | P1340 | -0.05 | 0.001 | 0.010 |
| 79 | ACVR1B | E497 | 0.09 | 0.001 | 0.012 |
| 80 | PRSS1 | P1249 | -0.05 | 0.001 | 0.012 |
| 81 | VAV2 | P1182 | -0.09 | 0.001 | 0.012 |
| 82 | ACTG2 | E98 | -0.08 | 0.001 | 0.012 |
| 83 | NRAS | P12 | 0.07 | 0.001 | 0.012 |
| 84 | PTPRG | E40 | 0.07 | 0.001 | 0.012 |
| 85 | ERBB4 | P541 | 0.10 | 0.001 | 0.012 |
| 86 | FGF7 | P610 | -0.06 | 0.001 | 0.012 |
| 87 | IFNG | P188 | -0.07 | 0.001 | 0.012 |
| 88 | WNT8B | P216 | -0.05 | 0.001 | 0.012 |
| 89 | NDN | P1110 | -0.06 | 0.001 | 0.012 |
| 90 | ITK | P114 | -0.08 | 0.001 | 0.012 |
| 91 | PADI4 | P1158 | -0.11 | 0.002 | 0.013 |
| 92 | PGR | P790 | -0.06 | 0.002 | 0.013 |
| 93 | HLA-DRA | P77 | 0.11 | 0.002 | 0.013 |
| 94 | BCL2L2 | P280 | 0.10 | 0.002 | 0.013 |
| 95 | CDH11 | P354 | 0.10 | 0.002 | 0.013 |
| 96 | DIO3 | P674 | 0.14 | 0.002 | 0.013 |
| 97 | USP29 | P282 | -0.04 | 0.002 | 0.013 |
| 98 | FAT | P279 | 0.08 | 0.002 | 0.013 |
| 99 | CD81 | P272 | 0.10 | 0.002 | 0.013 |
| 100 | SEMA3C | E49 | 0.09 | 0.002 | 0.014 |
| ^a^Change in average beta per decade | | |  |  |  |
|  |  |  |  |  |  |
| Pleura |  |  |  |  |  |
| Rank | *GENE* | CpG | Coefficient^a^ | *P -*value | *Q* - value |
| 1 | *HOXA9* | E252 | 0.50 | 1.00E-06 | 1.50E-05 |
| 2 | *CHGA* | E52 | 0.49 | 1.00E-06 | 2.50E-05 |
| 3 | *HOXA9* | P1141 | 0.46 | 1.00E-06 | 1.01E-04 |
| 4 | *FZD9* | E458 | 0.34 | 1.00E-06 | 1.20E-04 |
| 5 | *ASCL2* | P360 | 0.38 | 2.00E-06 | 3.84E-04 |
| 6 | *CD81* | P211 | 0.34 | 2.00E-06 | 3.84E-04 |
| 7 | *SOX17* | P303 | 0.39 | 2.00E-06 | 3.84E-04 |
| 8 | *MT1A* | E13 | 0.44 | 5.00E-06 | 0.001 |
| 9 | *DIO3* | E230 | 0.23 | 5.00E-06 | 0.001 |
| 10 | *SOX17* | P287 | 0.32 | 5.00E-06 | 0.001 |
| 11 | *SLC5A8* | E60 | 0.31 | 1.10E-05 | 0.001 |
| 12 | *PENK* | E26 | 0.33 | 1.10E-05 | 0.001 |
| 13 | *HOXA11* | P698 | 0.34 | 2.50E-05 | 0.003 |
| 14 | *HOXA9* | P303 | 0.27 | 2.60E-05 | 0.003 |
| 15 | *ASCL2* | P609 | 0.28 | 2.80E-05 | 0.003 |
| 16 | *PENK* | P447 | 0.33 | 3.10E-05 | 0.003 |
| 17 | *TAL1* | E122 | 0.35 | 4.70E-05 | 0.004 |
| 18 | *HS3ST2* | P171 | 0.35 | 5.90E-05 | 0.004 |
| 19 | *AATK* | P519 | 0.22 | 6.20E-05 | 0.004 |
| 20 | *TAL1* | P594 | 0.34 | 6.20E-05 | 0.004 |
| 21 | *HIC2* | P498 | 0.30 | 6.60E-05 | 0.004 |
| 22 | *IRF5* | E101 | 0.19 | 7.30E-05 | 0.004 |
| 23 | *GSTM1* | P266 | 0.24 | 7.50E-05 | 0.004 |
| 24 | *DLC1* | E276 | 0.27 | 8.00E-05 | 0.005 |
| 25 | *ASCL2* | E76 | 0.28 | 9.80E-05 | 0.005 |
| 26 | *HS3ST2* | E145 | 0.32 | 1.06E-04 | 0.005 |
| 27 | *CTNNB1* | P757 | 0.27 | 1.06E-04 | 0.005 |
| 28 | *AATK* | P709 | 0.22 | 1.36E-04 | 0.007 |
| 29 | *PAX6* | P1121 | 0.28 | 1.73E-04 | 0.008 |
| 30 | *HS3ST2* | P546 | 0.25 | 2.00E-04 | 0.009 |
| 31 | *TAL1* | P817 | 0.21 | 2.70E-04 | 0.012 |
| 32 | *EGR4* | P479 | 0.40 | 2.74E-04 | 0.012 |
| 33 | *MT1A* | P49 | 0.44 | 2.95E-04 | 0.012 |
| 34 | *HOXA11* | E35 | 0.30 | 3.30E-04 | 0.013 |
| 35 | *IPF1* | P750 | 0.26 | 3.46E-04 | 0.013 |
| 36 | *ALOX12* | P223 | 0.25 | 3.55E-04 | 0.013 |
| 37 | *TNFRSF10C* | E109 | 0.28 | 4.23E-04 | 0.016 |
| 38 | *CDKN2B* | S294 | 0.20 | 4.69E-04 | 0.017 |
| 39 | *CD81* | P272 | 0.17 | 0.001 | 0.021 |
| 40 | *FLT3* | E326 | 0.38 | 0.001 | 0.023 |
| 41 | *DNAJC15* | E26 | 0.17 | 0.001 | 0.028 |
| 42 | *PYCARD* | P150 | 0.13 | 0.001 | 0.030 |
| 43 | *NPY* | E31 | 0.24 | 0.001 | 0.034 |
| 44 | *JAK3* | P156 | 0.26 | 0.001 | 0.037 |
| 45 | *DDIT3* | P1313 | 0.23 | 0.001 | 0.039 |
| 46 | *JAK3* | E64 | 0.32 | 0.001 | 0.041 |
| 47 | *NGFR* | E328 | 0.26 | 0.002 | 0.045 |
| 48 | *MC2R* | P1025 | -0.15 | 0.002 | 0.057 |
| 49 | *CALCA* | E174 | 0.25 | 0.002 | 0.057 |
| 50 | *DES* | E228 | 0.20 | 0.002 | 0.058 |
| 51 | *CASP8* | E474 | -0.19 | 0.002 | 0.062 |
| 52 | *SLC5A5* | E60 | 0.17 | 0.002 | 0.062 |
| 53 | *EYA4* | E277 | 0.29 | 0.002 | 0.062 |
| 54 | *SOX1* | P1018 | 0.33 | 0.003 | 0.063 |
| 55 | *GABRA5* | P1016 | -0.19 | 0.003 | 0.063 |
| 56 | *KCNQ1* | E349 | 0.32 | 0.003 | 0.063 |
| 57 | *ALOX12* | E85 | 0.23 | 0.003 | 0.063 |
| 58 | *IGF2AS* | P203 | 0.21 | 0.003 | 0.063 |
| 59 | *CD40* | E58 | 0.31 | 0.003 | 0.064 |
| 60 | *CTSD* | P726 | -0.13 | 0.003 | 0.068 |
| 61 | *RAB32* | P493 | 0.19 | 0.003 | 0.068 |
| 62 | *IL16* | P93 | -0.17 | 0.003 | 0.074 |
| 63 | *TNFRSF1A* | P678 | 0.11 | 0.003 | 0.075 |
| 64 | *SOX1* | P294 | 0.28 | 0.004 | 0.085 |
| 65 | *FZD9* | P175 | 0.23 | 0.004 | 0.085 |
| 66 | *MOS* | E60 | 0.26 | 0.005 | 0.098 |
| 67 | *EMR3* | E61 | -0.18 | 0.005 | 0.099 |
| 68 | *TFF2* | P178 | -0.15 | 0.005 | 0.099 |
| 69 | *MSH2* | P1008 | 0.12 | 0.005 | 0.100 |
| 70 | *HCK* | P858 | 0.19 | 0.005 | 0.102 |
| 71 | *CDKN2A* | E121 | 0.12 | 0.006 | 0.113 |
| 72 | *RET* | P717 | 0.17 | 0.006 | 0.113 |
| 73 | *TNFRSF10C* | P7 | 0.20 | 0.006 | 0.113 |
| 74 | *CALCA* | P171 | 0.13 | 0.006 | 0.113 |
| 75 | *SYK* | E372 | 0.26 | 0.006 | 0.117 |
| 76 | *FLT1* | P615 | 0.30 | 0.007 | 0.124 |
| 77 | *HCK* | P46 | 0.31 | 0.007 | 0.126 |
| 78 | *THBS2* | P605 | 0.19 | 0.008 | 0.132 |
| 79 | *CREB1* | P819 | -0.16 | 0.008 | 0.132 |
| 80 | *KCNQ1* | P546 | 0.14 | 0.008 | 0.136 |
| 81 | *PTPRH* | P255 | -0.19 | 0.008 | 0.138 |
| 82 | *PLXDC2* | E337 | -0.15 | 0.009 | 0.144 |
| 83 | *CSF3* | E242 | 0.08 | 0.009 | 0.144 |
| 84 | *BSG* | P211 | 0.29 | 0.009 | 0.144 |
| 85 | *CD40* | P372 | 0.23 | 0.009 | 0.151 |
| 86 | *PRSS1* | E45 | -0.12 | 0.010 | 0.153 |
| 87 | *BCL2A1* | P1127 | 0.09 | 0.010 | 0.153 |
| 88 | *HOXB2* | P99 | 0.15 | 0.010 | 0.156 |
| 89 | *IL18BP* | E285 | 0.09 | 0.010 | 0.156 |
| 90 | *EYA4* | P508 | 0.20 | 0.011 | 0.160 |
| 91 | *GABRG3* | E123 | -0.17 | 0.012 | 0.179 |
| 92 | *MYBL2* | P354 | 0.25 | 0.012 | 0.179 |
| 93 | *FANCE* | P356 | 0.24 | 0.013 | 0.189 |
| 94 | *GSTM2* | E153 | 0.24 | 0.013 | 0.189 |
| 95 | *RASSF1* | P244 | 0.35 | 0.013 | 0.189 |
| 96 | *PWCR1* | P357 | -0.12 | 0.014 | 0.198 |
| 97 | *TWIST1* | P355 | 0.20 | 0.014 | 0.198 |
| 98 | *GFI1* | P45 | 0.25 | 0.015 | 0.204 |
| 99 | *DLK1* | E227 | 0.22 | 0.016 | 0.217 |
| 100 | *SFTPC* | E13 | -0.12 | 0.016 | 0.218 |
| ^a^Change in average beta per decade | | |  |  |  |
|  |  |  |  |  |  |
| Brain |  |  |  |  |  |
| Rank | *GENE* | CpG | Coefficient^a^ | *P -*value | *Q* - value |
| 1 | *PTHLH* | P757 | 0.21 | 1.00E-06 | 3.00E-06 |
| 2 | *ABCC5* | P444 | -0.18 | 1.00E-06 | 9.30E-05 |
| 3 | *RIPK2* | E123 | -0.26 | 1.00E-06 | 1.19E-04 |
| 4 | *ERCC1* | P440 | -0.44 | 1.00E-06 | 1.19E-04 |
| 5 | *JAG2* | P264 | -0.21 | 1.00E-06 | 2.02E-04 |
| 6 | *ERCC3* | P1210 | 0.13 | 1.10E-05 | 0.002 |
| 7 | *ONECUT2* | P315 | -0.50 | 3.50E-05 | 0.005 |
| 8 | *FASTK* | P257 | -0.28 | 4.30E-05 | 0.005 |
| 9 | *NPR2* | P618 | -0.44 | 6.70E-05 | 0.008 |
| 10 | *EPHX1* | P22 | -0.21 | 8.70E-05 | 0.009 |
| 11 | *EYA4* | E277 | -0.36 | 1.12E-04 | 0.010 |
| 12 | *TCF7L2* | E411 | -0.16 | 1.15E-04 | 0.010 |
| 13 | *MYOD1* | P50 | -0.51 | 1.26E-04 | 0.010 |
| 14 | *IRF5* | P123 | -0.24 | 2.56E-04 | 0.019 |
| 15 | *CCKAR* | P270 | 0.15 | 2.87E-04 | 0.020 |
| 16 | *H19* | P1411 | 0.21 | 4.51E-04 | 0.028 |
| 17 | *ERBB3* | E331 | 0.34 | 4.62E-04 | 0.028 |
| 18 | *IL10* | P348 | 0.18 | 0.001 | 0.034 |
| 19 | *NEFL* | P209 | -0.25 | 0.001 | 0.041 |
| 20 | *KLK10* | P268 | -0.32 | 0.001 | 0.065 |
| 21 | *PTGS2* | P308 | -0.20 | 0.001 | 0.065 |
| 22 | *SIN3B* | P514 | 0.16 | 0.001 | 0.066 |
| 23 | *SEMA3C* | P642 | 0.30 | 0.002 | 0.070 |
| 24 | *IGF2* | E134 | -0.72 | 0.002 | 0.070 |
| 25 | *PRKCDBP* | E206 | -0.43 | 0.002 | 0.071 |
| 26 | *PTHLH* | P15 | 0.15 | 0.002 | 0.074 |
| 27 | *KIAA0125* | E29 | 0.11 | 0.002 | 0.084 |
| 28 | *TPEF* | S88 | -0.16 | 0.002 | 0.084 |
| 29 | *ACVR2B* | E27 | -0.08 | 0.003 | 0.087 |
| 30 | *TEK* | E75 | -0.26 | 0.003 | 0.087 |
| 31 | *ALPL* | P278 | -0.28 | 0.003 | 0.087 |
| 32 | *ERCC1* | P354 | -0.11 | 0.003 | 0.087 |
| 33 | *HDAC9* | P137 | -0.29 | 0.003 | 0.087 |
| 34 | *ADAMTS12* | E52 | -0.35 | 0.003 | 0.089 |
| 35 | *AOC3* | P890 | -0.25 | 0.003 | 0.092 |
| 36 | *CCNA1* | E7 | 0.89 | 0.003 | 0.094 |
| 37 | *EPHB3* | E0 | -0.25 | 0.004 | 0.103 |
| 38 | *WRN* | E57 | -0.39 | 0.004 | 0.104 |
| 39 | *PAX6* | E129 | 0.33 | 0.004 | 0.104 |
| 40 | *CTSH* | E157 | -0.11 | 0.004 | 0.105 |
| 41 | *FRK* | P36 | -0.51 | 0.005 | 0.125 |
| 42 | *TAL1* | P817 | 0.25 | 0.005 | 0.126 |
| 43 | *COL18A1* | P365 | -0.31 | 0.006 | 0.126 |
| 44 | *NFKB2* | P709 | -0.35 | 0.006 | 0.126 |
| 45 | *MYB* | P673 | 0.18 | 0.006 | 0.126 |
| 46 | *RAB32* | E314 | -0.31 | 0.006 | 0.126 |
| 47 | *C20orf47* | P225 | -0.08 | 0.006 | 0.127 |
| 48 | *TCF4* | P317 | 0.54 | 0.006 | 0.128 |
| 49 | *HOXB13* | P17 | -0.34 | 0.006 | 0.128 |
| 50 | *THBS1* | P500 | 0.65 | 0.006 | 0.128 |
| 51 | *CD44* | P87 | -0.37 | 0.007 | 0.130 |
| 52 | *CASP10* | P334 | -0.40 | 0.007 | 0.130 |
| 53 | *NDN* | P1110 | 0.09 | 0.007 | 0.130 |
| 54 | *JUNB* | P1149 | 0.12 | 0.007 | 0.130 |
| 55 | *CTSH* | P238 | -0.18 | 0.007 | 0.130 |
| 56 | *GABRA5* | P1016 | 0.13 | 0.007 | 0.130 |
| 57 | *WEE1* | P924 | -0.42 | 0.007 | 0.130 |
| 58 | *HTR1B* | E232 | 0.33 | 0.007 | 0.130 |
| 59 | *WNT2* | P217 | -0.10 | 0.007 | 0.130 |
| 60 | *TM7SF3* | P1068 | 0.28 | 0.008 | 0.137 |
| 61 | *TNFRSF10D* | E27 | -0.49 | 0.009 | 0.149 |
| 62 | *TFDP1* | P543 | 0.11 | 0.009 | 0.150 |
| 63 | *CSTB* | E410 | -0.07 | 0.009 | 0.151 |
| 64 | *PDGFB* | E25 | 0.44 | 0.010 | 0.157 |
| 65 | *HDAC1* | P414 | 0.31 | 0.010 | 0.157 |
| 66 | *AGXT* | E115 | 0.12 | 0.010 | 0.158 |
| 67 | *MYCL1* | P502 | -0.35 | 0.010 | 0.158 |
| 68 | *PAX6* | P1121 | 0.24 | 0.011 | 0.160 |
| 69 | *EFNB3* | E17 | 0.21 | 0.011 | 0.162 |
| 70 | *ABL2* | P459 | 0.38 | 0.011 | 0.162 |
| 71 | *DDB2* | P613 | 0.35 | 0.011 | 0.163 |
| 72 | *SEMA3A* | P343 | 0.88 | 0.012 | 0.163 |
| 73 | *KRT13* | P676 | 0.11 | 0.012 | 0.163 |
| 74 | *IGFBP3* | E65 | -0.11 | 0.012 | 0.163 |
| 75 | *AFF3* | P808 | 0.09 | 0.012 | 0.163 |
| 76 | *TGFB3* | E58 | -0.48 | 0.012 | 0.163 |
| 77 | *RAP1A* | P285 | 0.41 | 0.012 | 0.163 |
| 78 | *DDR2* | P743 | -0.50 | 0.013 | 0.167 |
| 79 | *IRF7* | P277 | -0.43 | 0.013 | 0.167 |
| 80 | *GNMT* | P197 | -0.25 | 0.013 | 0.168 |
| 81 | *MCAM* | P265 | 0.34 | 0.013 | 0.169 |
| 82 | *PTPN6* | P282 | 0.11 | 0.014 | 0.175 |
| 83 | *IGFBP3* | P1035 | 0.44 | 0.014 | 0.179 |
| 84 | *PTPNS1* | E433 | -0.23 | 0.015 | 0.184 |
| 85 | *IHH* | P246 | -0.30 | 0.015 | 0.184 |
| 86 | *GJB2* | E43 | 0.30 | 0.015 | 0.184 |
| 87 | *MCC* | E23 | -0.19 | 0.016 | 0.184 |
| 88 | *KCNQ1* | E349 | 0.08 | 0.016 | 0.187 |
| 89 | *PDGFRB* | E195 | -0.31 | 0.017 | 0.191 |
| 90 | *GAS1* | E22 | -0.06 | 0.017 | 0.193 |
| 91 | *DLL1* | P832 | -0.35 | 0.017 | 0.197 |
| 92 | *HOXB13* | E21 | 1.19 | 0.018 | 0.198 |
| 93 | *HPSE* | P29 | -0.21 | 0.018 | 0.198 |
| 94 | *WT1* | P853 | -0.28 | 0.019 | 0.203 |
| 95 | *PYCARD* | E87 | -0.37 | 0.020 | 0.211 |
| 96 | *LMO2* | P794 | 0.26 | 0.020 | 0.211 |
| 97 | *HBII-52* | P659 | 0.19 | 0.020 | 0.211 |
| 98 | *COMT* | E401 | -0.18 | 0.020 | 0.211 |
| 99 | *NOS2A* | E117 | -0.18 | 0.020 | 0.211 |
| 100 | *TJP1* | P326 | -0.22 | 0.021 | 0.211 |
| ^a^Change in average beta per decade | | |  |  |  |
|  |  |  |  |  |  |
| Lung |  |  |  |  |  |
| Rank | *GENE* | CpG | Coefficient^a^ | *P -*value | *Q* - value |
| 1 | *DNMT3B* | P352 | -0.13 | 1.30E-05 | 0.007 |
| 2 | *ERN1* | P809 | -0.12 | 1.40E-05 | 0.007 |
| 3 | *FZD9* | E458 | 0.15 | 8.30E-05 | 0.025 |
| 4 | *EPHX1* | P1358 | -0.17 | 1.39E-04 | 0.032 |
| 5 | *BCL6* | P248 | -0.12 | 2.25E-04 | 0.037 |
| 6 | *ESR1* | P151 | 0.17 | 2.64E-04 | 0.037 |
| 7 | *PTHR1* | P258 | -0.13 | 3.18E-04 | 0.037 |
| 8 | *FRZB* | P406 | 0.09 | 3.27E-04 | 0.037 |
| 9 | *JAK3* | E64 | -0.10 | 0.001 | 0.131 |
| 10 | *KLK11* | P103 | -0.10 | 0.001 | 0.135 |
| 11 | *IL8* | E118 | -0.11 | 0.002 | 0.138 |
| 12 | *ICA1* | P72 | 0.13 | 0.002 | 0.138 |
| 13 | *DIO3* | P674 | 0.15 | 0.002 | 0.138 |
| 14 | *ATP10A* | P524 | -0.12 | 0.002 | 0.146 |
| 15 | *ERCC1* | P440 | -0.12 | 0.002 | 0.146 |
| 16 | *ONECUT2* | E96 | 0.13 | 0.003 | 0.147 |
| 17 | *ACTG2* | P346 | -0.09 | 0.003 | 0.147 |
| 18 | *AHR* | P166 | -0.13 | 0.003 | 0.147 |
| 19 | *IL17RB* | E164 | 0.15 | 0.003 | 0.147 |
| 20 | *STK11* | P295 | -0.10 | 0.004 | 0.147 |
| 21 | *MET* | E333 | -0.16 | 0.004 | 0.147 |
| 22 | *DCC* | P177 | 0.15 | 0.004 | 0.147 |
| 23 | *TSP50* | P137 | 0.18 | 0.004 | 0.147 |
| 24 | *PADI4* | P1158 | -0.13 | 0.004 | 0.148 |
| 25 | *DBC1* | E204 | 0.12 | 0.004 | 0.148 |
| 26 | *ISL1* | P379 | 0.13 | 0.004 | 0.148 |
| 27 | *RASGRF1* | E16 | 0.10 | 0.004 | 0.148 |
| 28 | *NAT2* | P11 | -0.10 | 0.005 | 0.148 |
| 29 | *RARA* | P1076 | 0.15 | 0.005 | 0.153 |
| 30 | *PADI4* | P1011 | -0.10 | 0.005 | 0.153 |
| 31 | *TNFRSF10C* | P7 | 0.09 | 0.005 | 0.153 |
| 32 | *PITX2* | E24 | 0.12 | 0.006 | 0.165 |
| 33 | *ADAMTS12* | P250 | -0.07 | 0.006 | 0.169 |
| 34 | *MYH11* | P236 | 0.11 | 0.006 | 0.169 |
| 35 | *EPHB6* | P827 | -0.05 | 0.007 | 0.178 |
| 36 | *EVI1* | P30 | 0.09 | 0.007 | 0.178 |
| 37 | *CDKN2A* | S188 | 0.09 | 0.007 | 0.178 |
| 38 | *SH3BP2* | P771 | -0.07 | 0.007 | 0.178 |
| 39 | *FES* | P223 | 0.15 | 0.008 | 0.178 |
| 40 | *BMPR1A* | P956 | 0.11 | 0.008 | 0.178 |
| 41 | *ITK* | P114 | -0.10 | 0.008 | 0.178 |
| 42 | *PGR* | P790 | -0.07 | 0.008 | 0.178 |
| 43 | *RASGRF1* | P768 | -0.08 | 0.009 | 0.180 |
| 44 | *BCL3* | E71 | -0.06 | 0.010 | 0.198 |
| 45 | *EPHA3* | P106 | 0.13 | 0.011 | 0.229 |
| 46 | *MUSK* | P308 | -0.09 | 0.012 | 0.229 |
| 47 | *WNT5A* | P655 | 0.08 | 0.012 | 0.229 |
| 48 | *MATK* | P64 | 0.10 | 0.012 | 0.229 |
| 49 | *CDKN2B* | S294 | 0.12 | 0.012 | 0.232 |
| 50 | *EPHA7* | E6 | 0.07 | 0.013 | 0.234 |
| 51 | *PLG* | E406 | -0.07 | 0.013 | 0.234 |
| 52 | *GPX1* | P194 | -0.08 | 0.014 | 0.234 |
| 53 | *DBC1* | P351 | 0.18 | 0.014 | 0.234 |
| 54 | *PROM1* | P44 | -0.09 | 0.014 | 0.234 |
| 55 | *ERBB3* | P870 | 0.13 | 0.015 | 0.234 |
| 56 | *IGF1* | P933 | -0.11 | 0.015 | 0.234 |
| 57 | *NOS2A* | P288 | -0.10 | 0.015 | 0.234 |
| 58 | *FN1* | E469 | 0.10 | 0.016 | 0.234 |
| 59 | *TBX1* | P885 | 0.15 | 0.016 | 0.234 |
| 60 | *SOX2* | P546 | 0.10 | 0.017 | 0.234 |
| 61 | *MMP3* | P16 | -0.06 | 0.017 | 0.234 |
| 62 | *FRZB* | E186 | 0.14 | 0.017 | 0.234 |
| 63 | *WNT8B* | E487 | -0.07 | 0.017 | 0.234 |
| 64 | *PDGFB* | P719 | -0.07 | 0.018 | 0.234 |
| 65 | *MMP19* | E274 | -0.09 | 0.018 | 0.234 |
| 66 | *HLA-DRA* | P132 | -0.09 | 0.018 | 0.234 |
| 67 | *EPHA8* | P256 | -0.06 | 0.018 | 0.234 |
| 68 | *BCL2L2* | E172 | 0.10 | 0.018 | 0.234 |
| 69 | *CSK* | P740 | 0.08 | 0.019 | 0.234 |
| 70 | *FGFR1* | P204 | -0.06 | 0.019 | 0.234 |
| 71 | *MMP2* | P197 | 0.11 | 0.020 | 0.234 |
| 72 | *HS3ST2* | E145 | 0.14 | 0.020 | 0.234 |
| 73 | *CSF1R* | P73 | -0.06 | 0.020 | 0.234 |
| 74 | *HBII-52* | P563 | -0.06 | 0.020 | 0.234 |
| 75 | *TRPM5* | P979 | -0.11 | 0.021 | 0.234 |
| 76 | *DES* | P1006 | -0.07 | 0.021 | 0.234 |
| 77 | *KDR* | P445 | 0.11 | 0.021 | 0.234 |
| 78 | *FLT3* | E326 | 0.07 | 0.021 | 0.234 |
| 79 | *CDKN1B* | P1161 | -0.10 | 0.021 | 0.234 |
| 80 | *B3GALT5* | P330 | -0.06 | 0.021 | 0.234 |
| 81 | *ID1* | P880 | -0.06 | 0.022 | 0.234 |
| 82 | *PRSS1* | P1249 | -0.07 | 0.022 | 0.234 |
| 83 | *COL18A1* | P365 | 0.05 | 0.022 | 0.234 |
| 84 | *DAB2* | P468 | 0.09 | 0.022 | 0.234 |
| 85 | *LIF* | P383 | -0.08 | 0.022 | 0.234 |
| 86 | *PALM2-AKAP2* | P183 | 0.06 | 0.022 | 0.234 |
| 87 | *SLC22A3* | E122 | 0.16 | 0.023 | 0.234 |
| 88 | *APBA2* | P305 | -0.05 | 0.023 | 0.234 |
| 89 | *LOX* | P313 | 0.11 | 0.023 | 0.234 |
| 90 | *EPHA3* | E156 | 0.09 | 0.023 | 0.234 |
| 91 | *DNMT1* | P100 | -0.08 | 0.023 | 0.234 |
| 92 | *PI3* | P1394 | -0.08 | 0.025 | 0.240 |
| 93 | *GRB7* | E71 | 0.12 | 0.025 | 0.240 |
| 94 | *SMARCA4* | P362 | -0.07 | 0.025 | 0.240 |
| 95 | *AOC3* | P890 | -0.07 | 0.026 | 0.240 |
| 96 | *AGTR1* | P154 | 0.22 | 0.026 | 0.240 |
| 97 | *SNRPN* | P230 | -0.06 | 0.026 | 0.240 |
| 98 | *FGF1* | E5 | -0.08 | 0.026 | 0.240 |
| 99 | *PTK7* | E317 | -0.05 | 0.027 | 0.240 |
| 100 | *SLIT2* | P208 | 0.12 | 0.027 | 0.240 |
| ^a^Change in average beta per decade | | |  |  |  |
|  |  |  |  |  |  |
| Head and Neck |  |  |  |  |  |
| Rank | *GENE* | CpG | Coefficient^a^ | *P -*value | *Q* - value |
| 1 | *LMO2* | E148 | 1.07 | 8.00E-05 | 0.000 |
| 2 | *IL10* | P348 | 0.65 | 8.80E-05 | 0.050 |
| 3 | *SIN3B* | P607 | 0.43 | 1.93E-04 | 0.050 |
| 4 | *TM7SF3* | P1068 | 0.68 | 2.17E-04 | 0.050 |
| 5 | *ABCB4* | P892 | -0.25 | 2.72E-04 | 0.050 |
| 6 | *AIM2* | P624 | 0.66 | 2.76E-04 | 0.050 |
| 7 | *HPN* | P823 | 0.70 | 3.21E-04 | 0.050 |
| 8 | *LMO2* | P794 | 0.77 | 3.41E-04 | 0.050 |
| 9 | *TNFSF8* | E258 | 0.72 | 3.54E-04 | 0.050 |
| 10 | *LTB4R* | P163 | 0.46 | 3.54E-04 | 0.050 |
| 11 | *PECAM1* | E32 | 0.71 | 4.36E-04 | 0.052 |
| 12 | *CARD15* | P665 | 0.50 | 4.40E-04 | 0.052 |
| 13 | *PECAM1* | P135 | 0.55 | 0.001 | 0.062 |
| 14 | *GADD45A* | P737 | 0.33 | 0.001 | 0.067 |
| 15 | *DNMT3B* | P352 | -0.35 | 0.001 | 0.087 |
| 16 | *GFI1* | P208 | 0.56 | 0.001 | 0.087 |
| 17 | *SMARCB1* | P220 | -0.75 | 0.001 | 0.092 |
| 18 | *SPI1* | P48 | 0.53 | 0.001 | 0.094 |
| 19 | *AFF3* | P122 | 0.64 | 0.001 | 0.099 |
| 20 | *ERCC3* | P1210 | 0.45 | 0.002 | 0.124 |
| 21 | *RAD54B* | P227 | -0.39 | 0.002 | 0.124 |
| 22 | *IL10* | P85 | 0.69 | 0.002 | 0.124 |
| 23 | *LTB4R* | E64 | 0.64 | 0.002 | 0.124 |
| 24 | *EVI2A* | P94 | 0.67 | 0.002 | 0.124 |
| 25 | *SOD3* | P225 | 0.41 | 0.002 | 0.133 |
| 26 | *ASB4* | P391 | -0.21 | 0.002 | 0.133 |
| 27 | *MET* | E333 | -0.49 | 0.003 | 0.134 |
| 28 | *MPL* | P62 | 0.48 | 0.003 | 0.142 |
| 29 | *THPO* | E483 | 0.40 | 0.003 | 0.145 |
| 30 | *DDB2* | P407 | -0.48 | 0.003 | 0.160 |
| 31 | *TEK* | P479 | -0.26 | 0.004 | 0.167 |
| 32 | *CDK10* | P199 | -0.31 | 0.004 | 0.167 |
| 33 | *MPO* | E302 | 0.33 | 0.004 | 0.167 |
| 34 | *HIC1* | S103 | 0.47 | 0.004 | 0.167 |
| 35 | *PADI4* | E24 | 0.58 | 0.004 | 0.181 |
| 36 | *TIE1* | E66 | 0.58 | 0.005 | 0.207 |
| 37 | *NCL* | P1102 | -0.18 | 0.006 | 0.218 |
| 38 | *OSM* | P34 | 0.54 | 0.006 | 0.218 |
| 39 | *PPAT* | E170 | 0.22 | 0.006 | 0.235 |
| 40 | *DDB2* | P613 | -0.53 | 0.010 | 0.337 |
| 41 | *CLK1* | P538 | -0.31 | 0.010 | 0.337 |
| 42 | *TAL1* | E122 | 0.29 | 0.010 | 0.337 |
| 43 | *39696* | P464 | -0.42 | 0.011 | 0.351 |
| 44 | *CPA4* | E20 | -0.46 | 0.011 | 0.357 |
| 45 | *RAD50* | P191 | -0.35 | 0.012 | 0.376 |
| 46 | *HOXA5* | E187 | -0.78 | 0.012 | 0.381 |
| 47 | *LAT* | E46 | 0.45 | 0.014 | 0.425 |
| 48 | *OSM* | P188 | 0.62 | 0.015 | 0.432 |
| 49 | *GP1BB* | P278 | 0.48 | 0.016 | 0.444 |
| 50 | *KRT13* | P676 | 0.28 | 0.016 | 0.444 |
| 51 | *TK1* | E47 | 0.09 | 0.017 | 0.465 |
| 52 | *SPI1* | E205 | 0.34 | 0.017 | 0.465 |
| 53 | *CD2* | P68 | 0.52 | 0.018 | 0.488 |
| 54 | *PSCA* | E359 | 0.28 | 0.019 | 0.491 |
| 55 | *HOXA5* | P1324 | -0.58 | 0.019 | 0.491 |
| 56 | *RHOH* | P121 | 0.68 | 0.020 | 0.503 |
| 57 | *IL1B* | P582 | 0.21 | 0.021 | 0.507 |
| 58 | *ITPR2* | P804 | 0.17 | 0.021 | 0.507 |
| 59 | *CSF3R* | P8 | 0.40 | 0.021 | 0.507 |
| 60 | *EMR3* | P1297 | 0.20 | 0.022 | 0.519 |
| 61 | *FGF7* | P44 | -0.27 | 0.023 | 0.529 |
| 62 | *HOXB2* | P99 | 0.55 | 0.024 | 0.537 |
| 63 | *CD34* | P780 | 0.51 | 0.024 | 0.540 |
| 64 | *MLH3* | E72 | 0.17 | 0.024 | 0.540 |
| 65 | *ABCC2* | P88 | -0.10 | 0.026 | 0.565 |
| 66 | *CDH17* | E31 | -0.16 | 0.028 | 0.591 |
| 67 | *PADI4* | P1158 | -0.58 | 0.029 | 0.611 |
| 68 | *CD34* | P339 | 0.43 | 0.029 | 0.611 |
| 69 | *TRPM5* | P721 | -0.18 | 0.031 | 0.619 |
| 70 | *IL12B* | P1453 | -0.24 | 0.032 | 0.619 |
| 71 | *ABCC2* | E16 | -1.72 | 0.033 | 0.619 |
| 72 | *ELL* | P693 | -0.20 | 0.033 | 0.619 |
| 73 | *CSF3R* | P472 | 0.52 | 0.033 | 0.619 |
| 74 | *WNT8B* | P216 | -0.15 | 0.033 | 0.619 |
| 75 | *IGF1* | P933 | -0.42 | 0.033 | 0.619 |
| 76 | *LEFTY2* | P561 | -0.24 | 0.033 | 0.619 |
| 77 | *P2RX7* | P597 | -0.21 | 0.036 | 0.653 |
| 78 | *PLAUR* | E123 | 0.14 | 0.036 | 0.656 |
| 79 | *PLAT* | E158 | -0.47 | 0.037 | 0.656 |
| 80 | *HSPA2* | P162 | -0.23 | 0.038 | 0.667 |
| 81 | *IL18BP* | P51 | 0.44 | 0.040 | 0.689 |
| 82 | *RARA* | P1076 | 0.61 | 0.040 | 0.689 |
| 83 | *CCL3* | E53 | 0.27 | 0.041 | 0.694 |
| 84 | *CRK* | P721 | -0.36 | 0.042 | 0.694 |
| 85 | *TSP50* | E21 | -0.29 | 0.042 | 0.694 |
| 86 | *HSD17B12* | P97 | 0.09 | 0.042 | 0.694 |
| 87 | *LIG3* | P622 | -0.49 | 0.043 | 0.694 |
| 88 | *MC2R* | P1025 | -0.35 | 0.045 | 0.718 |
| 89 | *AGXT* | E115 | -0.11 | 0.046 | 0.733 |
| 90 | *SMARCA4* | P362 | 0.13 | 0.047 | 0.737 |
| 91 | *EVI2A* | E420 | 0.38 | 0.047 | 0.737 |
| 92 | *TIMP2* | E394 | 0.15 | 0.048 | 0.742 |
| 93 | *CD86* | P3 | 0.39 | 0.049 | 0.742 |
| 94 | *USP29* | P205 | -0.06 | 0.050 | 0.744 |
| 95 | *SEMA3C* | P642 | 0.36 | 0.050 | 0.744 |
| 96 | *NAT2* | P11 | -0.36 | 0.051 | 0.744 |
| 97 | *TIAM1* | P117 | -0.19 | 0.051 | 0.748 |
| 98 | *TNFSF8* | P184 | 0.50 | 0.053 | 0.764 |
| 99 | *CSF1R* | E26 | 0.34 | 0.054 | 0.764 |
| 100 | *SPP1* | P647 | 0.25 | 0.054 | 0.764 |
| ^a^Change in average beta per decade | | |  |  |  |
|  |  |  |  |  |  |
| Blood |  |  |  |  |  |
| Rank | *GENE* | CpG | Coefficient^a^ | *P -*value | *Q* - value |
| 1 | *FZD9* | E458 | 0.32 | 1.38E-04 | 0.112 |
| 2 | *RARRES1* | P426 | -0.28 | 1.62E-04 | 0.112 |
| 3 | *JAK3* | P1075 | -0.27 | 2.39E-04 | 0.112 |
| 4 | *HOXB2* | P488 | -0.17 | 3.85E-04 | 0.126 |
| 5 | *ZNF264* | P397 | -0.34 | 4.66E-04 | 0.126 |
| 6 | *SPDEF* | P6 | -0.31 | 0.001 | 0.126 |
| 7 | *IL1B* | P582 | -0.12 | 0.001 | 0.126 |
| 8 | *EPHA2* | P203 | -0.18 | 0.001 | 0.128 |
| 9 | *IL12B* | E25 | -0.11 | 0.001 | 0.128 |
| 10 | *ACVR1* | E328 | -0.24 | 0.001 | 0.128 |
| 11 | *PCGF4* | P92 | 0.20 | 0.001 | 0.174 |
| 12 | *CTSD* | P726 | -0.25 | 0.001 | 0.174 |
| 13 | *DNAJC15* | P65 | -0.11 | 0.002 | 0.193 |
| 14 | *NBL1* | E205 | -0.29 | 0.002 | 0.241 |
| 15 | *ZP3* | P220 | -0.16 | 0.003 | 0.241 |
| 16 | *GSTM2* | P453 | -0.12 | 0.003 | 0.267 |
| 17 | *PXN* | P308 | -0.13 | 0.003 | 0.267 |
| 18 | *GRB7* | P160 | -0.21 | 0.004 | 0.319 |
| 19 | *ITPR3* | E86 | -0.08 | 0.005 | 0.319 |
| 20 | *KCNK4* | E3 | -0.14 | 0.005 | 0.319 |
| 21 | *IGFBP6* | E47 | -0.08 | 0.005 | 0.319 |
| 22 | *RAD54B* | P227 | -0.39 | 0.005 | 0.319 |
| 23 | *BCR* | P346 | -0.24 | 0.005 | 0.319 |
| 24 | *ZIM3* | P718 | -0.14 | 0.006 | 0.328 |
| 25 | *MYB* | P673 | -0.06 | 0.006 | 0.345 |
| 26 | *NPR2* | P1093 | -0.16 | 0.006 | 0.345 |
| 27 | *DDIT3* | P1313 | -0.17 | 0.007 | 0.347 |
| 28 | *TERT* | P360 | -0.06 | 0.007 | 0.371 |
| 29 | *HLA-DOB* | P357 | -0.09 | 0.008 | 0.409 |
| 30 | *SLC22A18* | P472 | -0.11 | 0.009 | 0.410 |
| 31 | *CHGA* | P243 | -0.25 | 0.009 | 0.410 |
| 32 | *SPP1* | P647 | -0.22 | 0.010 | 0.410 |
| 33 | *WNT1* | E157 | -0.12 | 0.010 | 0.410 |
| 34 | *SOD3* | P225 | -0.15 | 0.011 | 0.426 |
| 35 | *SLC22A18* | P216 | -0.19 | 0.011 | 0.426 |
| 36 | *PDGFB* | P719 | -0.19 | 0.011 | 0.426 |
| 37 | *HLA-DPA1* | P205 | -0.20 | 0.012 | 0.426 |
| 38 | *ABCA1* | E120 | -0.08 | 0.013 | 0.426 |
| 39 | *MAP3K1* | P7 | -0.08 | 0.013 | 0.426 |
| 40 | *ARHGAP9* | P260 | 0.04 | 0.013 | 0.426 |
| 41 | *CYP2E1* | P416 | 0.12 | 0.013 | 0.426 |
| 42 | *HLA-DPA1* | E35 | -0.17 | 0.014 | 0.426 |
| 43 | *TIE1* | E66 | -0.18 | 0.014 | 0.426 |
| 44 | *HOXB2* | P99 | -0.14 | 0.014 | 0.426 |
| 45 | *TWIST1* | P44 | -0.12 | 0.014 | 0.426 |
| 46 | *PLAT* | P80 | -0.20 | 0.014 | 0.426 |
| 47 | *FAS* | P322 | -0.26 | 0.015 | 0.453 |
| 48 | *PLSCR3* | P751 | -0.14 | 0.015 | 0.453 |
| 49 | *PTGS1* | E80 | -0.14 | 0.018 | 0.485 |
| 50 | *ERCC3* | P1210 | -0.15 | 0.018 | 0.485 |
| 51 | *GSTM1* | P363 | -0.32 | 0.019 | 0.485 |
| 52 | *IL17RB* | E164 | -0.18 | 0.019 | 0.485 |
| 53 | *FGFR3* | P1152 | -0.23 | 0.019 | 0.485 |
| 54 | *SEMA3F* | P692 | -0.13 | 0.019 | 0.485 |
| 55 | *CSF1* | P339 | -0.09 | 0.019 | 0.485 |
| 56 | *SEMA3B* | P110 | -0.16 | 0.020 | 0.485 |
| 57 | *CARD15* | P302 | -0.29 | 0.020 | 0.485 |
| 58 | *IL17RB* | P788 | -0.14 | 0.020 | 0.485 |
| 59 | *CSF1* | P217 | -0.08 | 0.021 | 0.485 |
| 60 | *AXL* | P223 | -0.21 | 0.021 | 0.485 |
| 61 | *CSF3* | E242 | -0.14 | 0.022 | 0.485 |
| 62 | *MAPK4* | E273 | 0.08 | 0.022 | 0.485 |
| 63 | *PTK6* | E50 | -0.13 | 0.022 | 0.485 |
| 64 | *EPHA2* | P340 | -0.14 | 0.022 | 0.485 |
| 65 | *BCR* | P422 | -0.17 | 0.022 | 0.485 |
| 66 | *MMP19* | E274 | -0.09 | 0.023 | 0.485 |
| 67 | *SLC14A1* | E295 | -0.09 | 0.024 | 0.496 |
| 68 | *TNFRSF1A* | P678 | -0.15 | 0.024 | 0.502 |
| 69 | *CCND3* | P435 | -0.14 | 0.025 | 0.502 |
| 70 | *LTB4R* | P163 | -0.16 | 0.026 | 0.502 |
| 71 | *CRIP1* | P874 | -0.15 | 0.026 | 0.502 |
| 72 | *TFPI2* | P152 | -0.09 | 0.027 | 0.502 |
| 73 | *ASCL1* | P747 | -0.17 | 0.027 | 0.502 |
| 74 | *DDR1* | P332 | -0.14 | 0.027 | 0.502 |
| 75 | *SNCG* | E119 | -0.13 | 0.027 | 0.502 |
| 76 | *CSF3* | P309 | -0.13 | 0.027 | 0.502 |
| 77 | *RBL2* | P250 | -0.25 | 0.027 | 0.502 |
| 78 | *ASCL1* | E24 | -0.10 | 0.028 | 0.513 |
| 79 | *STK11* | P295 | -0.18 | 0.029 | 0.515 |
| 80 | *PECAM1* | E32 | -0.16 | 0.029 | 0.515 |
| 81 | *ERN1* | P809 | -0.14 | 0.030 | 0.515 |
| 82 | *LRRK1* | P834 | -0.07 | 0.031 | 0.532 |
| 83 | *TMEFF1* | P626 | -0.17 | 0.032 | 0.532 |
| 84 | *SERPINE1* | E189 | -0.12 | 0.032 | 0.532 |
| 85 | *PWCR1* | P811 | -0.13 | 0.034 | 0.558 |
| 86 | *OGG1* | E400 | -0.16 | 0.035 | 0.558 |
| 87 | *PYCARD* | P393 | -0.08 | 0.035 | 0.558 |
| 88 | *AOC3* | P890 | -0.10 | 0.035 | 0.558 |
| 89 | *CARD15* | P665 | -0.15 | 0.036 | 0.558 |
| 90 | *ZAP70* | P220 | -0.11 | 0.036 | 0.558 |
| 91 | *MMP14* | P13 | -0.14 | 0.037 | 0.558 |
| 92 | *ASCL2* | E76 | 0.06 | 0.037 | 0.558 |
| 93 | *PECAM1* | P135 | -0.15 | 0.037 | 0.558 |
| 94 | *ERCC1* | P440 | -0.23 | 0.038 | 0.561 |
| 95 | *IL10* | P348 | -0.15 | 0.038 | 0.561 |
| 96 | *TSG101* | P139 | -0.09 | 0.039 | 0.561 |
| 97 | *HSPA2* | P162 | -0.06 | 0.039 | 0.561 |
| 98 | *SPI1* | P48 | -0.16 | 0.039 | 0.564 |
| 99 | *GRB10* | P496 | -0.14 | 0.041 | 0.581 |
| 100 | *SNCG* | P53 | -0.22 | 0.041 | 0.581 |
| ^a^Change in average beta per decade | | |  |  |  |
