# Supplementary material for: Aging and Environmental Exposures Alter Tissue-Specific DNA Methylation Dependent upon CpG Island Context
Source: PLoS Genet. 2009 Aug 14;5(8):e1000602. doi: 10.1371/journal.pgen.1000602 (PMC2718614; doi:10.1371/journal.pgen.1000602)
Supplement: Table S7 — Pyrosequencing assay primers. (0.05 MB DOC) [file pgen.1000602.s009.doc]

| Table S7. Pyrosequencing assay primers. | |  |  |  |  |  |
| --- | --- | --- | --- | --- | --- | --- |
| GENE | Primer | Tm | %GC | Amplicon bp | CpGs analyzed | Score^a^ |
| *RARA_*P176 |  |  |  |  |  |  |
| Forward | ATGTTGTGTATTTGAGATAGGGAATTGT | 70.0 | 32.1 |  |  |  |
| Reverse^b^ | CACTCCCCAACTAAAAAACTCTCC | 70.3 | 45.8 |  |  |  |
| Sequencing | TGTATTTGAGATAGGGAATT | 50.7 | 30.0 | 170 | 6 | 89 |
| *DNMT3B*_P352 |  |  |  |  |  |  |
| Forward | GGAGATTTTGGTGTTGTGTGATTA | 69.0 | 37.5 |  |  |  |
| Reverse^b^ | TTTTTCCCCAACTCCTTTCA | 68.4 | 40.0 |  |  |  |
| Sequencing | GGTTTTGTTTTTTTTGAG | 50.0 | 27.8 | 108 | 3 | 78 |
| *LIF*_P383 |  |  |  |  |  |  |
| Forward^b^ | TGGTAGAGTTAGTAGGGGTTTTAGTAGTG | 68.5 | 41.4 |  |  |  |
| Reverse | ACACAAAAACCAATCTCCATCTACTAAT | 69.4 | 32.1 |  |  |  |
| Sequencing | CAAAAACCAATCTCCAT | 35.3 | 35.3 | 109 | 3 | 81 |
| *FZD9*_E458 |  |  |  |  |  |  |
| Forward | GTGTCGCGGTATCGGTTATAAT | 68.9 | 45.5 |  |  |  |
| Reverse^b^ | AACGCAAATAACTATAACAACCGTACTAC | 69.0 | 34.5 |  |  |  |
| Sequencing | AGGCGGTTGTCGAGT | 55.0 | 60.0 | 131 | 5 | 90 |
| ^a^PSQ Assay Design Software score; ^b^Biotin labeled | |  |  |  |  |  |
